# Differentiation of oral bacteria in *in vitro* cultures and human saliva by secondary electrospray ionization – mass spectrometry

**DOI:** 10.1038/srep15163

**Published:** 2015-10-19

**Authors:** Lukas Bregy, Annick R. Müggler, Pablo Martinez-Lozano Sinues, Diego García-Gómez, Yannick Suter, Georgios N. Belibasakis, Malcolm Kohler, Patrick R. Schmidlin, Renato Zenobi

**Affiliations:** 1 Department of Chemistry and Applied Biosciences, ETH Zürich, Switzerland; 2 Clinic of Preventive Dentistry, Periodontology and Cariology, Center of Dental Medicine, University of Zürich, Switzerland; 3 Oral Microbiology and Immunology, Institute of Oral Biology, Center of Dental Medicine, University of Zürich Switzerland; 4 Department of Pulmonology, University Hospital Zürich, Switzerland

## Abstract

The detection of bacterial-specific volatile metabolites may be a valuable tool to predict infection. Here we applied a real-time mass spectrometric technique to investigate differences in volatile metabolic profiles of oral bacteria that cause periodontitis. We coupled a secondary electrospray ionization (SESI) source to a commercial high-resolution mass spectrometer to interrogate the headspace from bacterial cultures and human saliva. We identified 120 potential markers characteristic for periodontal pathogens *Aggregatibacter actinomycetemcomitans* (n = 13), *Porphyromonas gingivalis* (n = 70), *Tanerella forsythia* (n = 30) and *Treponema denticola* (n = 7) in *in vitro* cultures. In a second proof-of-principle phase, we found 18 (*P. gingivalis*, *T. forsythia* and *T. denticola*) of the 120 *in vitro* compounds in the saliva from a periodontitis patient with confirmed infection with *P. gingivalis*, *T. forsythia* and *T. denticola* with enhanced ion intensity compared to two healthy controls. In conclusion, this method has the ability to identify individual metabolites of microbial pathogens in a complex medium such as saliva.

Periodontal bacterial infections are one of the most severe dental diseases, often even leading to tooth loss if untreated^1^. The disease is characterized by a progressive damage of the periodontal soft and hard tissue and is frequently accompanied by four types of bacteria that can colonize the mouth: *Aggregatibacter actinomycetemcomitans*, *Porphyromonas gingivalis*, *Tanerella forsythia* and *Treponema denticola*. Often, patients suffering of periodontitis only show indicating symptoms at a late stage, when the development of the disease has led to enhanced tooth mobility.

Today, dentists are identifying this disease mainly by visual inspections of the teeth, looking for specific signs of inflammation like bleeding upon probing and increased periodontal pocket depths^2^. In addition, bacteria samples from periodontal pockets can nowadays be analyzed using microbiological techniques to identify potential disease related bacterial species^3^. However there is still a lack of methods with the combined power of fast, sensitive and specific analysis of such bacteria. Early on, it was recognized that saliva is an ideal fluid to analyze the processes in the mouth and it is being used for clinical diagnostics. Saliva contains many different compound classes, including small molecules, proteins or enzymes, which also means that it contains rich information related to processes taking place in the oral cavity^4^.

In the last decades, mass spectrometry has been shown to be a powerful tool to analyze biological samples like blood, urine, saliva, and breath, for applications in clinical chemistry and in toxicology^5^,^6^,^7^. Traditionally, hyphenated methods like gas chromatography – mass spectrometry (GC-MS) or liquid chromatography – mass spectrometry (LC-MS) were used to analyze the headspace and the culture solution of bacteria respectively^8^. After some pioneering developments^9^,^10^,^11^,^12^, several real-time techniques for the analysis of gas and vapor samples were developed in recent years, e.g., proton transfer reaction – mass spectrometry (PTR-MS) and selected ion flow tube – mass spectrometry (SIFT-MS). A similarly powerful analytical tool is secondary electrospray ionization – mass spectrometry (SESI-MS), where vapor species are ionized at atmospheric pressure and are subsequently detected by any commercial mass spectrometer of choice. It has shown promise in a number of applications calling for fast and sensitive analysis of vapors^13^,^14^,^15^,^16^. It has been extensively used for the analysis of volatile metabolic “fingerprints”, including bacterial species^17^,^18^,^19^,^20^,^21^. A key element is that, with minor modifications, one can take advantage of the power of modern mass spectrometers, especially their high resolving power, sensitivity and MSMS capabilities. This is crucial in real-time analysis because, in the absence of prior chromatographic separation, metabolite detection and accurate identification relies exclusively in MS performance. As a result, SESI combined with high performance MS results in rich breathprints covering volatiles and semi-volatiles, e.g., fatty acids^22^.

Following the idea of fast, sensitive and selective diagnostics, e.g., in dentistry, we show here the first untargeted headspace analyses of oral bacteria *A. actinomycetemcomitans*, *P. gingivalis*, *T. denticola* and *T. forsythia* with high-resolution SESI-MS. In a first *in vitro* part of our study, we analyzed the headspace of 5 independent biological replicate cultures from each bacterium. In a second phase, we tested whether the set of molecules found to be discriminatory in the *in vitro* study could also be found in saliva samples from one periodontitis patient.

## Results and Discussion

### Untargeted bacteria culture headspace analysis

**Figure 1:**
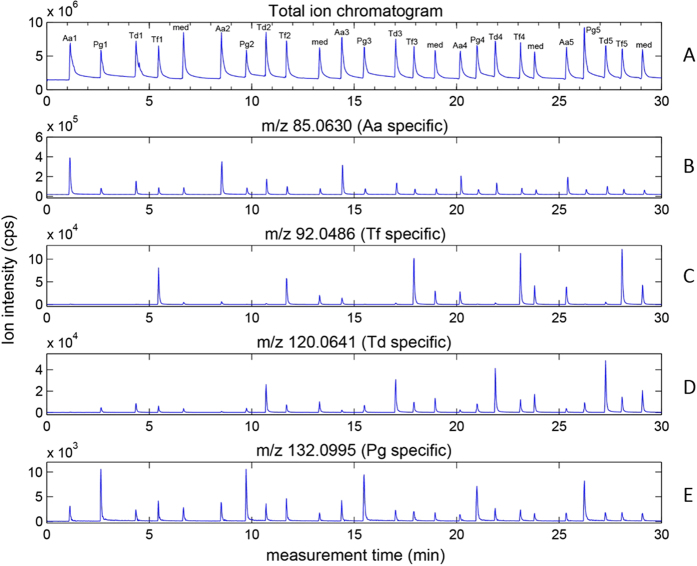
Headspace analysis showing the total ion chromatogram (A) and extracted ion chromatograms of four bacteria specific compounds (B - E): m/z 85.0630 (A. *actinomycetemcomitans* specific), m/z 92.0486 (T. *forsythia* specific), m/z 120.0641 (T. *denticola* specific) and m/z 132.0995 (P. *gingivalis* specific).

Figure 1 shows that SESI-MS is a suitable real-time method to analyze volatiles accumulated in the headspace of bacteria medium. The total ion current from headspace injections of the four different bacteria strains and of the mixed medium is shown (total of 25 measurements; Panel A). Note how the total intensity rises sharply during the injection of the gas sample and decays within ~1 minute to the baseline level. Also of note is that the 25 mass spectrometric measurements were completed within 30 minutes, without any sample pretreatment. Extracted ion time-profiles for four selected mass peaks are plotted (B-E). Importantly, the intensities of the biological replicates are in most cases comparable, with the exception of the first *T. denticola* biological replicate at m/z 120.0641, which shows a lower intensity. By mere visual inspection of the four m/z time-profiles alone, one can already easily distinguish that each compound exhibit a different intensity in each of the bacterial strains investigated.

**Figure 2:**
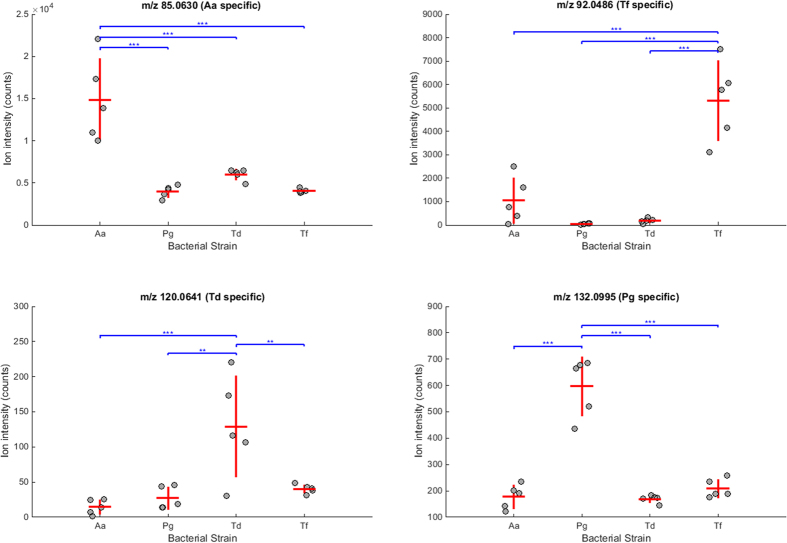
Plots showing the ion intensities of the headspace samples from all four bacteria cultures for four selected compounds m/z 85.0630 (Aa—*A. actinomycetemcomitans* specific), m/z 92.0486 (Tf – *T. forsythia* specific), m/z 120.0641 (Td – *T. denticola* specific) and m/z 132.0995 (Pg – *P. gingivalis* specific). The horizontal red line represents the mean, while the vertical red line indicate standard deviation. The blue brackets connecting the boxes indicate a significant difference between the biological replicates originating from two bacterial strains. The number of stars indicate the range of p-values from the multiple comparison test (Tukey-Kramer procedure): * (0.01< p ≤ 0.05), ** (0.001 < p ≤ 0.01) and *** (p ≤ 0.001).

Figure 2 displays the data distribution per group for the selected features shown in Fig. 1. It can be noticed that the intensities of the biological replicates from one bacterial strain are significantly enhanced in comparison to the other three strains. With the exception of the *T. denticola* outlier for m/z 120.0641, these four volatiles alone could distinguish the four strains investigated. It is visible that the ion intensities for the non-specific strains is not equal to zero. This is reasonable, since it is unlikely that these metabolites are completely unique to one strain, but rather likely that they are produced in higher concentrations for a specific strain.

Apart from these four examples, the volatile fingerprints of the bacterial strains revealed a total of 120 bacteria-specific compounds (Table 1): 13 for *A. actinomycetemcomitans*, 70 for *P. gingivalis*, 7 for *T. denticola* and 30 for *T. forsythia*. The large number of *P. gingivalis, A. actinomycetemcomitans* and *T. forsythia*-specific compounds makes it very distinct, in contrast to *T. denticola*. Table 1 lists the molecular formulas of these compounds, along with p- and q-values. Supplementary Tables S1 and S2 provide further details on actual ion intensities for all bacterial cultures and culture medium; and pairwise comparisons, respectively.

**Figure 3:**
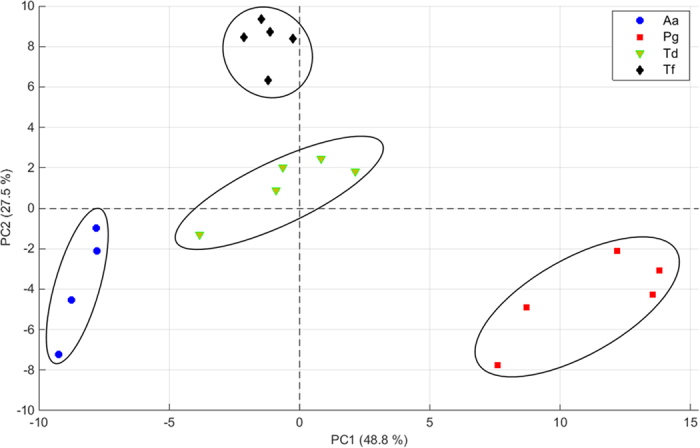
Projection of the mass spectra from the biological replicates of the four different bacteria strains *A. actinomycetemcomitans* (circles), *P. gingivalis* (squares), *T. denticola* (triangles) and *T. forsythia* (rhomboids) onto a two-dimensional PCA subspace. The replicates are clustering and the strains distinguishable from each other.

For a better visualization, the 120 filtered mass features from all biological replicates from the four different bacteria strains were subjected to principal component analysis (PCA). Figure 3 shows the score plot for the first two principal components, explaining ~76% of the variance. It can be observed that the five biological replicates per strain tend to cluster together and each bacterial type occupies a distinct area. The first PC separates *P. gingivalis* and *A. actinomycetemcomitans* from the rest, whereas PC 2 separates *T. forsythia* from the other three strains. With the combined information of these two axes it is possible to differentiate all four bacterial strains.

### Targeted bacteria analysis in human saliva

In the second phase of this study, the set of bacteria-specific compounds found *in vitro* (Table 1, Tables S1 and S2) were sought in saliva samples from one patient and two healthy controls. The patient suffered a severe periodontitis, and *P. gingivalis*, *T. denticola* and *T. forsythia* bacteria were present. The number of bacteria was determined by the standard IAI Pado-Test 4.5: 0.45 ± 0.9 E6 Pg, 0.28 ± 0.53 E6 Td and 0.51 ± 1.02 E6 Tf bacteria (mean of the number of bacteria in four dental pockets). It has to be stated that only in 2 of 4 pockets the bacteria were present, although all pockets had a periodontal screening index equal to 4, which is the highest possible score for the disease. As expected, none of the four oral bacteria strains were found in the dental pockets of the controls. Out of the 120 bacteria-specific compounds identified *in vitro*, 94 were found to be present in saliva. This is consistent with previous studies showing that the transfer of potential markers from *in vitro* to *in vivo* is not always possible, due to different conditions such as different media^23^. Remarkably, 18 of these compounds were systematically present with enhanced ion intensities in the patient in comparison to the healthy controls. 13 compounds were related to *P. gingivalis*, 4 to *T. forsythia* and 1 to *T. denticola*. These compounds were systematically enhanced in the patient’s saliva as compared to the two controls (average patient/control ratio 4.7 and 4.6 for both controls). Supplementary Table S3 lists ion intensities and patient/controls ratios for each of the 120 molecules of interest. While the number of participants in this study is limited, the fact that 18 molecules are systematically detected both in *in vitro* cultures and enhanced in the saliva of a patient suffering periodontitis, supports the hypothesis that pathogen-related volatiles could be used as indicators of periodontitis development.

## Conclusions

We have shown that with simple modifications of the atmospheric pressure interface of commercial mass spectrometers, a rapid screening of volatiles found in the headspace of bacterial cultures and saliva is feasible. SESI-MS produced rich mass spectrometric fingerprints of volatiles with masses up to >200 Da. In addition, the high accuracy and high mass resolution of the MS systems used in this study enabled us to provide molecular formulae of the bacteria-related chemicals with high confidence.

During the initial headspace analysis of pure bacterial cultures, we were able to differentiate four oral bacteria strains: *A. actinomycetemcomitans*, *P. gingivalis*, *T. denticola*, and *T. forsythia*. The 120 most discriminative compounds found *in vitro* were then used for targeted analysis of the saliva samples from a severe periodontitis patient and two healthy controls. As a result, we found a set of 18 compounds highly increased in the saliva of the patient as compared to the controls. We conclude that this method has potential for clinical diagnosis of bacterial infections in the oral cavity such as periodontitis. Follow-up measurements with a larger cohort of patients and healthy controls should be accomplished to validate these preliminary results and to correlate the absolute number of oral bacteria with the volatile compounds abundance.

## Methods

### Bacteria cultures

*Aggregatibacter actinomycetemcomitans* and *P. gingivalis* were cultivated on Colombia Blood Agar (CBA) plates and afterwards used to inoculate 10 mL of a Brain Heart Infusion (BHI) liquid medium, under aerobic (*A. actinomycetemcomitans*) or anaerobic (*P. gingivalis*) conditions, at 37 °C. In the next step, 5% of the liquid culture was sub-cultivated under the same conditions for 24 hours. *Treponema denticola* was cultivated under anaerobic conditions in 10 mL of spirochetes medium OMIZ-W68^24^ for 5 days, and thereafter 10% of this volume were sub-cultured into the same medium and cultivated anaerobically at 37 °C for 8 days. *Tannerella forsythia* was cultivated under anaerobic conditions for 3 days. Thereafter, 10% volume was transferred in modified^25^ spirochetes medium OMIZ-W68^24^, and thereafter sub-cultured anaerobically at 37 °C for 3 days. All bacteria suspensions were adjusted to an optical density (OD) _550_ _nm_ = 0.5 and centrifuged at 4’200 rpm (3’600 g) for 10 minutes, at 4 °C. The supernatants were finally sterilized by filtration (pore diameter 0.2 μm), transferred into 20 mL glass vials with septa (Infochroma, Zug, Switzerland) and stored at −20 °C until further use. From each bacterial strain, five biological replicates were produced. It is important to note the bacteria were cultured *in vitro* in different specific media: BHI for *A. actinomycetemcomitans and P. gingivalis;* OMIZ-W68 for *T. denticola* and modified OMIZ-W68 for *T. forsythia*. To counteract artefact volatiles resulting from different media, the media were pooled prior headspace analysis.

### Human subjects and standard oral bacteria tests

All three study participants were non-smoking male volunteers. One patient with severe periodontitis and two healthy controls were selected for the explorative targeted analysis of the human saliva samples. The participants were examined for their periodontitis status by a dentist. During a periodontal basic examination (PGU) the periodontal screening index (PSI) in six dental areas was measured. The criteria to be patient was to have a PSI of four (periodontal pocket depth deeper than 5.5 mm) in at least two of the dental sextants. A healthy control should have a maximum PSI of one (periodontal pocket depth not deeper than 3.5 mm) in all sextants. In addition, the absolute number of bacteria for all four strains (*A. actinomycetemcomitans*, *P. gingivalis*, *T. denticola*, *T. forsythia*) was determined in four periodontal pockets (teeth no. 16, 25, 36 and 46) with the commercially available IAI Pado-Test 4.5 (IAI AG, Zuchwil, Switzerland). The ethical committee of the Kanton Zürich (KEK, Stampfenbachstrasse 121, 8090 Zürich) approved the experiments (KEK-ZH-Nr. 2013–0353) and all volunteers gave written informed consent to participate. All experiments were carried out in accordance with the approved protocol.

## Sample preparation

### Bacteria cultures

In the cultivation experiments three different media (BHI, OMIZ-W86, modified OMIZ-W86) were used. To avoid the assignment of media compounds as potential bacteria strain markers, all samples were spiked with the two remaining media. For example, 100 μL of the Aa or Pg samples were spiked with 100 μL OMIZ-W86 and 100 μL modified OMIZ-W86 medium, and likewise for the other two media. And 100 μL of each Tf sample was spiked with 100 μL BHI and 100 μL OMIZ-W86 medium. After vortexing, the sample vials were flushed with pressurized air (medicinal air, Pangas, Dagmersellen, Switzerland) with a flow rate of 2 L min^−1^.

### Human saliva samples

From all test subjects 1–2 mL saliva were sampled into 20 mL glass vials (Infochroma, Zug, Switzerland) after they had not drunk, eaten, smoked or cleaned their teeth for one hour. The samples were stored at −18 °C between sampling and analysis. Before the measurements, the sample vials were brought to room temperature and flushed with pressurized air (medicinal air, Pangas, Dagmersellen, Switzerland) with a flow rate of 2 L min^−1^.

### Secondary electrospray ionization – mass spectrometry (SESI-MS)

**Figure 4:**
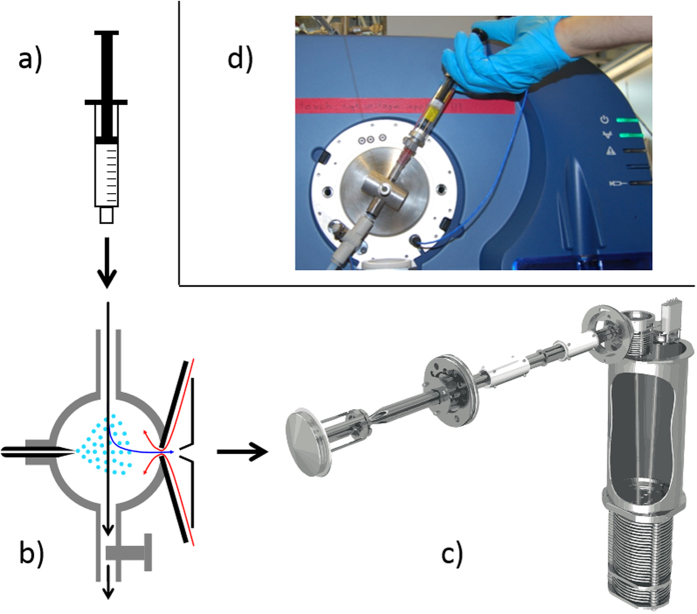
(**a**) Gas tight syringe with a headspace sample from the bacteria cultures (**b**) SESI reaction chamber with nano electrospray mounted coaxially with the mass spectrometer inlet. The sample was injected into the chamber, where secondary ionization takes place. (**c**) Quadrupole time-of-flight hybrid mass spectrometer for analyzing the ionized headspace samples in real-time. (**d**) Schematic diagram of the sample introduction system into the SESI-MS system. Photos are reproduced with permission, courtesy of AB Sciex Pte. Ltd.

For this type of metabolomic analysis, a quadrupole time-of-flight instrument was chosen because of its high resolution and sensitivity. We interfaced a home-built SESI source with a TripleTOF 5600^+^ mass spectrometer (10’000 resolution at m/z 40 to 32’000 resolution at m/z 450/Applied Biosystems Sciex, Toronto, ON, Canada/Fig. 4). The standard ESI source was removed, the SESI source was installed on the “curtain plate” and the original curtain gas was replaced by an auxiliary gas supply (2.4 L min^−1^ of high purity nitrogen, heated to 60 °C). The SESI source consisted of a cylindrical stainless steel reaction chamber with two observation windows (glass), two inlets (nano electrospray and sample delivery) and one outlet (backpressure vent). Coaxially with the inlet of the mass spectrometer, an uncoated fused silica capillary (id 20 μm, TaperTip Emitters, New Objectives, Woburn, MA, USA) was fixed in the chamber wall to establish a nano electrospray. The spray was pneumatically (approx. 500 mbar overpressure of air) supplied with nanopure water (resistivity 18.2 MΩ cm, Barnstead Nanopure, Thermo Fisher Scientific, Waltham, MA, USA) and 0.1% formic acid (98%, for MS, Fluka, Sigma-Aldrich, Buchs, Switzerland) as solvent. To establish the nanoES, high voltage (3.6 kV) was taken from the mass spectrometer and applied to the solvent reservoir via a platinum wire. The nanoES was optically and electrically checked by a microscope (Specwell) and a multimeter (Uni-Trend, China). The spray current was optimized to 60–80 nA. The backpressure vent (Legris, Parker, Mesa, AZ, USA) was optimized for maximum signal intensity while introducing air with an overpressure of 10 mbar into the reaction chamber.

For analysis, 10 mL headspace were extracted from the sample vials with a gas tight syringe (10 mL, Hamilton, Reno, NV, USA) and injected into the reaction chamber were secondary ionization by the nano-electrospray took place. For each bacterial strain and human subject, a clean syringe was used. The mass spectrometer was acquiring mass spectra (m/z 40–450) in positive ionization mode with an accumulation time of 1 s.

### Compound identification

For further identification of the found biomarker with sum formula determination, the SESI source described for the TripleTOF 5600^+^ was adapted to a LTQ Orbitrap high-resolution mass analyzer (Thermo Fisher Scientific, Waltham, MA, USA) that has a resolution of 300’000 at m/z 60 to 100’000 at m/z 400. 100 μL of all 4 *in vivo* cultured bacteria samples spiked with the different media were mixed together and flushed with pressurized air (medicinal air, Pangas, Dagmersellen, Switzerland) with a flow rate of 2 L min^−1^. Afterwards 10 mL headspace were extracted and injected into the SESI – Orbitrap mass analyzer. The mass spectrometer was acquiring mass spectra (m/z 50–450) in positive ionization mode with an accumulation time of 1 s. Based on the exact mass sum formulae were provided based on the seven golden rules for sum formula determination by mass spectrometry^26^.

## Data Analysis

### Data pre-processing

The data was acquired and mass calibrated with the Analyst TF 1.7 and PeakView 2.1 software (Applied Biosystems Sciex, Toronto, ON, Canada). The mass spectrometric spectra of all bacteria culture supernatant and individual medium sample injections were exported as txt files. All subsequent data analysis was done with Matlab 2014a (MathWorks, Natick, MA, USA). (*i*) All the spectra were resampled using a linear interpolation function (2’000’000 data points across the 40–450 m/z range); (*ii*) to remove systematic variation between spectra, we applied median normalization; (*iii*) The spectra were centroided. An intensity threshold of 50 counts was set resulting in 1,966 features. Subsequently, signals that rise with time upon sample injection were identified. As a result, 547 of the 1’966 features were retained for further data analysis.

### Statistical analysis

The next step was to filter out the most informative features to discriminate one bacterial strain from the others. We pursued a univariate approach for this task. Thus, a comparison of differences in each of the detected compounds in the headspace of the bacterial samples was assessed by ANOVA or Kruskal-Wallis tests for normally and non-normally distributed data, respectively. Normality of the data was evaluated using a Lilliefors test. It followed a pairwise comparison by using a Tukey-Kramer procedure. Statistical significance was set at p-value ≤ 0.05. The false discovery rate for multiple hypothesis testing was estimated using the procedure introduced by Storey^27^.

This procedure delivered 464 statistically significant features. We further selected those which had significantly low intensity for three bacterial strains and an enhanced intensity for one bacterial strain which means that the p-values (from multiple comparison) between the specific strain and the other strains had to be lower than 0.05. In addition, the feature intensity in the medium sample had to be lower than in the bacteria samples. This procedure reduced the list of discriminative features to 149. A closer inspection of these features revealed the presence of redundant ^13^C isotopes, finally reducing the list to 120 discriminatory signals. Further dimensionality reduction was accomplished by subjecting the normalized 20 x 120 (samples x features) matrix to a principal component analysis (PCA) for better visualization of the data. For the targeted saliva analysis, the ion intensities of the 120 *in vitro* signals were compared between the patient and the two healthy controls. An arbitrary 2-fold intensity enhancement cut-off value was used to determine whether the compounds were enhanced in the saliva of the patient vs. the healthy controls.

## Additional Information

**How to cite this article**: Bregy, L. *et al*. Differentiation of oral bacteria in *in vitro* cultures and human saliva by secondary electrospray ionization – mass spectrometry. *Sci. Rep*. **5**, 15163; doi: 10.1038/srep15163 (2015).

## Supplementary Material

Supplementary Information

## Figures and Tables

**Table 1:** List of 120 discriminative compounds for *in vitro* bacteria cultures of *A. actinomycetemcomitans* (n = 13), *P. gingivalis* (n = 70), *T. denticola* (n = 7) and *T. forsythia* (n = 30).

Bacterial strain	m/z	Sum formula	Mass deviation from experimental mass (ppm)	*in vitro* comparisons		Present in patient’s saliva (IAI Pado-Test 4.5)	Enhanced in patient vs. controls	Enhancement ratio	
				p value	q value			patient/control 1	patient/control 2
Aa	43.0180	C2H3O	3.7	1.93E-05	1.32E-07	no	not present	**–**	**–**
Aa	58.8706	–	–	1.85E-03	4.13E-06	no	no	0.18	0.15
Aa	58.9993	–	–	2.04E-03	4.50E-06	no	not present	–	–
Aa	59.0134	C2H3O2	11	3.32E-03	6.59E-06	no	not present	–	–
Aa	59.0481	C3H7O	11	1.21E-03	3.11E-06	no	no	0.22	0.15
Aa	65.0369	C5H5	26	3.35E-05	1.97E-07	no	yes	27.42	88.89
Aa	67.0522	C5H7	1.9	8.09E-05	3.87E-07	no	no	0.43	0.31
Aa	84.8031	–	–	1.24E-03	3.13E-06	no	not present	–	–
Aa	85.0630	C5H9O	3.4	8.50E-06	7.04E-08	no	no	0.50	0.42
Aa	87.0667	C2H7N4	2	3.18E-04	1.16E-06	no	not present	–	–
Aa	99.0778	C6H11O	12	1.92E-04	7.69E-07	no	no	0.33	0.18
Aa	117.0886	C6H13O2	1.8	4.28E-04	1.43E-06	no	no	1.23	1.33
Aa	144.1094	C6H14N3O4	24	1.29E-04	5.71E-07	no	no	0.82	0.86
**Pg**	**79.0189**	**C5H3O**	**1.8**	**6.18E-04**	**1.91E-06**	**yes**	**yes**	**3.28**	**4.65**
Pg	88.0738	C4H10NO	2.2	7.25E-08	2.20E-09	yes	no	1.55	2.21
Pg	97.0258	C5H5O2	0.1	6.27E-06	5.50E-08	yes	no	0.34	0.30
Pg	97.0619	C6H9O	0.9	1.48E-09	2.17E-10	yes	no	0.47	0.48
Pg	100.0450	C4H6NO2	57	1.03E-04	4.61E-07	yes	no	1.15	1.23
**Pg**	**107.0669**	**C4H11O3**	**31**	**9.38E-04**	**2.64E-06**	**yes**	**yes**	**3.06**	**3.53**
Pg	110.0579	C6H8NO	19	3.39E-04	1.19E-06	yes	no	1.13	1.40
Pg	111.0414	C6H7O2	3.2	8.31E-08	2.33E-09	yes	no	0.37	0.38
Pg	111.0771	C7H11O	0.2	8.71E-09	4.82E-10	yes	no	1.02	1.12
Pg	113.0573	C6H9O2	1.8	3.02E-07	5.55E-09	yes	no	0.94	1.38
Pg	115.0355	C5H7O3	23	7.75E-04	2.29E-06	yes	no	0.20	0.23
Pg	115.0730	C6H11O2	0.2	2.48E-07	4.87E-09	yes	no	0.75	0.85
Pg	116.0498	C8H6N	9.3	1.00E-06	1.23E-08	yes	no	0.62	0.44
Pg	116.0680	C5H10NO2	1.8	3.62E-09	3.11E-10	yes	no	0.84	1.08
Pg	121.0274	C7H5O2	8.3	1.42E-05	1.07E-07	yes	no	1.19	1.26
Pg	125.0568	C7H9O2	3.2	4.59E-05	2.50E-07	yes	no	0.33	0.40
Pg	125.0931	C8H13O	24	4.84E-07	7.30E-09	yes	no	1.37	1.55
Pg	127.0732	C7H11O2	2	9.02E-09	4.82E-10	yes	no	0.60	0.81
Pg	128.0754	C3H6N5O	2.2	1.30E-09	2.17E-10	yes	no	0.67	0.84
**Pg**	**128.1042**	**C7H14NO**	**0.2**	**1.11E-07**	**2.97E-09**	**yes**	**yes**	**2.41**	**3.03**
Pg	129.0511	C2H5N6O	1	7.85E-06	6.60E-08	yes	no	0.68	0.68
Pg	129.0881	C7H13O2	0.8	2.28E-07	4.63E-09	yes	no	0.77	0.83
**Pg**	**130.0611**	**C9H8N**	**3.3**	**6.46E-06**	**5.51E-08**	**yes**	**yes**	**2.50**	**2.67**
Pg	130.0838	C6H12NO2	2.7	3.04E-04	1.11E-06	yes	no	1.21	1.68
Pg	132.0995	C6H14NO2	3.1	1.61E-08	7.90E-10	yes	no	1.34	1.73
Pg	134.0785	C5H12NO3	2.8	9.89E-04	2.71E-06	yes	not present	–	–
Pg	136.0722	C8H10NO	21	2.11E-05	1.39E-07	yes	no	0.93	1.32
**Pg**	**137.0216**	**C7H5O3**	**7.9**	**1.13E-05**	**8.99E-08**	**yes**	**yes**	**2.35**	**2.75**
**Pg**	**138.0143**	**C6H4NO3**	**31**	**6.63E-05**	**3.33E-07**	**yes**	**yes**	**2.49**	**2.89**
Pg	140.1120	C8H14NO	31	1.70E-06	1.96E-08	yes	no	0.55	0.64
Pg	141.0990	C8H13O2	2.9	2.54E-04	9.63E-07	yes	not present	–	–
Pg	143.1035	C8H15O2	1.8	1.18E-05	9.23E-08	yes	no	0.87	0.80
Pg	144.1349	C8H18NO	4.1	7.70E-05	3.73E-07	yes	no	1.75	2.51
Pg	145.0988	C6H13N2O2	1.8	9.63E-04	2.68E-06	yes	no	0.41	0.43
Pg	147.0421	C9H7O2	2.4	8.27E-04	2.42E-06	yes	no	0.27	0.31
**Pg**	**148.0756**	**C9H10NO**	**0.6**	**7.67E-04**	**2.28E-06**	**yes**	**yes**	**4.07**	**4.39**
Pg	149.0780	C6H13O4	2.2	5.36E-04	1.71E-06	yes	no	1.27	2.02
Pg	153.0607	C8H9O3	2.1	1.06E-03	2.87E-06	yes	not present	–	–
Pg	155.1042	C9H15O2	1.7	6.00E-06	5.35E-08	yes	no	0.60	0.92
Pg	157.1191	C9H17O2	1.9	3.70E-09	3.11E-10	yes	no	0.56	0.65
Pg	158.0824	C7H12NO3	13	2.31E-05	1.51E-07	yes	not present	–	–
**Pg**	**158.1515**	**C9H20NO**	**17**	**2.82E-04**	**1.06E-06**	**yes**	**yes**	**7.06**	**8.68**
**Pg**	**159.1093**	**C7H15N2O2**	**2.5**	**2.63E-05**	**1.63E-07**	**yes**	**yes**	**2.04**	**2.96**
Pg	162.0949	C7H16NOS	1.2	3.69E-04	1.28E-06	yes	not present	–	–
Pg	163.0716	C6H7N6	2.3	9.74E-05	4.41E-07	yes	no	0.63	0.76
**Pg**	**165.0992**	**C10H13O2**	**4.3**	**6.82E-03**	**1.20E-05**	**yes**	**yes**	**2.06**	**2.76**
Pg	171.0982	C5H11N6O	0.5	5.19E-05	2.73E-07	yes	no	0.94	1.33
Pg	171.1351	C6H15N6	1	1.29E-10	3.80E-11	yes	no	0.64	0.79
Pg	171.1461	C9H19N2O	2.3	7.37E-08	2.20E-09	yes	no	1.91	2.42
Pg	172.1664	C10H22NO	2.3	2.57E-03	5.41E-06	yes	no	1.65	1.82
Pg	175.1297	C5H15N6O	1.1	1.39E-04	5.97E-07	yes	no	1.98	2.21
Pg	179.0607	C9H11N2S	3.6	6.86E-05	3.42E-07	yes	no	1.06	1.69
Pg	179.1042	C7H11N6	1	1.26E-06	1.51E-08	yes	no	0.48	0.59
Pg	182.1148	C10H16NO2	15	1.97E-05	1.32E-07	yes	not present	–	–
Pg	185.1506	C11H21O2	3.8	4.34E-07	7.09E-09	yes	no	0.47	0.53
**Pg**	**189.1455**	**C10H21O3**	**3.8**	**1.37E-03**	**3.34E-06**	**yes**	**yes**	**2.34**	**2.52**
Pg	191.1394	C13H19O	3.4	1.46E-07	3.43E-09	yes	no	0.67	0.88
Pg	192.1645	C9H22NO3	26	6.97E-05	3.42E-07	yes	not present	–	–
Pg	193.1416	C9H21O4	3.8	3.64E-05	2.12E-07	yes	no	3.10	0.91
Pg	195.1349	C12H19O2	4.9	2.13E-04	8.45E-07	yes	no	0.60	0.67
Pg	199.1299	C7H15N6O	8.5	3.30E-04	1.18E-06	yes	no	0.62	0.88
Pg	199.1676	C12H23O2	1.3	8.04E-07	1.06E-08	yes	no	1.15	1.28
Pg	201.1095	C10H17O4	4.2	4.80E-07	7.30E-09	yes	no	1.59	2.37
**Pg**	**204.1399**	**C13H18NO**	**7.9**	**5.05E-09**	**3.30E-10**	**yes**	**yes**	**3.35**	**4.18**
Pg	205.1409	C10H21O4	3.6	2.29E-04	8.82E-07	yes	no	1.30	2.33
Pg	206.1358	C9H20NO4	14	2.28E-06	2.48E-08	yes	not present	–	–
Pg	214.0884	C10H16NO2S	3.4	1.90E-04	7.69E-07	yes	no	0.18	0.22
Pg	235.1863	C15H23O2	3.6	2.60E-05	1.62E-07	yes	not present	–	–
Pg	236.1563	C10H22NO5	30	8.80E-05	4.08E-07	yes	not present	–	–
**Pg**	**251.1842**	**C12H27O5**	**2.8**	**1.55E-04**	**6.45E-07**	**yes**	**yes**	**7.77**	**7.74**
Td	119.0570	C7H7N2	28	1.84E-04	7.57E-07	yes	not present	–	–
Td	120.0641	C4H10NO3	1.8	7.35E-04	2.22E-06	yes	no	0.02	0.03
Td	120.0796	C3H10N3O2	11	5.00E-04	1.63E-06	yes	no	0.67	0.41
**Td**	**121.0819**	**C5H13O3**	**3.5**	**4.33E-04**	**1.44E-06**	**yes**	**yes**	**2.47**	**2.36**
Td	136.1085	C9H14N	2	7.73E-05	3.73E-07	yes	no	0.53	1.09
Td	136.1299	–	–	1.18E-03	3.06E-06	yes	no	0.02	0.03
Td	150.1237	C5H16N3O2	25	2.55E-03	5.40E-06	yes	no	0.57	0.60
Tf	44.0492	C2H6N	6.2	6.46E-05	3.30E-07	yes	not present	–	–
Tf	50.0164	–	–	9.77E-04	2.70E-06	yes	not present	–	–
Tf	53.0378	C4H5	15	5.14E-04	1.65E-06	yes	not present	–	–
Tf	55.0279	C2H3N2	21	4.49E-09	3.30E-10	yes	not present	–	–
Tf	55.0530	C4H7	24	5.54E-08	2.04E-09	yes	no	0.65	0.52
Tf	60.0801	C3H10N	1.3	3.22E-05	1.92E-07	yes	no	0.55	0.92
Tf	70.0716	–	–	4.08E-07	6.86E-09	yes	not present	–	–
Tf	73.0441	C2H5N2O	43	8.28E-07	1.06E-08	yes	no	0.22	0.22
**Tf**	**73.0623**	**C4H9O**	**33**	**1.30E-07**	**3.19E-09**	**yes**	**yes**	**3.37**	**2.97**
Tf	76.0809	–	–	1.57E-07	3.56E-09	yes	not present	–	–
Tf	77.0362	C6H5	31	7.41E-07	1.04E-08	yes	no	0.37	0.31
Tf	79.0515	C6H7	34	2.72E-07	5.17E-09	yes	no	0.34	0.27
Tf	87.0785	C5H11O	13	6.23E-08	2.16E-09	yes	no	1.51	1.09
Tf	89.0577	C4H9O2	2.3	4.73E-04	1.54E-06	yes	no	1.31	1.19
Tf	92.0486	C6H6N	9.5	6.21E-07	8.91E-09	yes	no	0.20	0.07
Tf	96.0859	C6H10N	53	9.65E-05	4.40E-07	yes	no	0.49	0.47
**Tf**	**100.0180**	**C2H3N2O2**	**38**	**3.23E-04**	**1.16E-06**	**yes**	**yes**	**5.12**	**2.79**
Tf	103.0630	C2H7N4O	13	1.75E-05	1.23E-07	yes	not present	–	–
Tf	103.0729	C5H11O2	1.5	1.77E-06	2.01E-08	yes	no	0.64	0.60
Tf	107.0463	C2H7N2O3	14	3.30E-07	5.88E-09	yes	no	0.29	0.29
Tf	109.0516	C3H9O4	19	4.33E-05	2.42E-07	yes	not present	–	–
Tf	109.0981	C8H13	2.5	5.06E-06	4.73E-08	yes	no	1.26	0.81
Tf	115.1081	C7H15O	3	2.78E-08	1.17E-09	yes	no	0.98	0.66
Tf	116.1120	C6H14NO	43	4.00E-04	1.36E-06	yes	not present	–	–
Tf	123.1131	C9H15	1.8	5.57E-06	5.04E-08	yes	no	0.29	0.43
**Tf**	**127.1082**	**C8H15O**	**1.1**	**1.70E-05**	**1.20E-07**	**yes**	**yes**	**12.17**	**8.27**
**Tf**	**142.0290**	**C9H4NO**	**1.8**	**4.13E-12**	**2.43E-12**	**yes**	**yes**	**17.36**	**13.13**
Tf	142.0474	C6H8NO3	17	2.52E-05	1.59E-07	yes	not present	–	–
Tf	183.1711	C12H23O	4.6	1.65E-05	1.19E-07	yes	no	0.31	0.32
Tf	189.1261	C13H17O	6.8	7.80E-04	2.29E-06	yes	no	0.78	0.59

Compounds in bold were found at least a factor of two more intense in the saliva of the patient vs. two controls.
