# Supplementary material for: Differentiation of oral bacteria in in vitro cultures and human saliva by secondary electrospray ionization – mass spectrometry
Source: Sci Rep. 2015 Oct 19;5:15163. doi: 10.1038/srep15163 (PMC4609958; doi:10.1038/srep15163)
Supplement: Supplementary Information [file srep15163-s1.doc]

**SUPPLEMENTARY MATERIAL FOR**

**Differentiation of oral bacteria in *in vitro* cultures and human saliva by secondary electrospray ionization – mass spectrometry**

Lukas Bregy^1^, Annick R. Müggler^2^, Pablo Martinez-Lozano Sinues^1^, Diego Garcia Gomez^1^, Yannick Suter^1^, Georgios N. Belibasakis^3^, Malcolm Kohler^4^, Patrick R. Schmidlin^2^, and Renato Zenobi^1*^

**^1^**Department of Chemistry and Applied Biosciences, ETH Zürich, Switzerland, **^2^**Clinic of Preventive Dentistry, Periodontology and Cariology, Center of Dental Medicine, University of Zürich, Switzerland, **^3^**Oral Microbiology and Immunology, Institute of Oral Biology, Center of Dental Medicine, University of Zürich Switzerland, **^4^**Department of Pulmonology, University Hospital Zürich, Switzerland

*to whom correspondence should be addressed: [zenobi@org.chem.ethz.ch](mailto:zenobi@org.chem.ethz.ch)

The following Supplementary material accompanies this paper:

Table S1. Biomarkers for differentiating the four dental bacteria strains Aa, Pg, Td and Tf.

Table S2. List containing all statistical key values.

Table S3. List with the results of the targeted application of the *in vitro* found biomarkers towards human saliva samples from one periodontitis patient and two healthy controls.

**Table S1.** Found biomarkers to differentiate the four dental bacteria strains Aa, Pg, Td and Tf. For the elucidation of the sum formula the experimental mass from the Orbitrap - MS measurement was used if the deviation from the Q-TOF mass was minor than 30 ppm. Otherwise the Q-TOF - MS feature mass was used.

| **Bacteria strain specifity** | **m/z measured by QTOF-MS** | **m/z measured by Orbitrap - MS** | **Sum Formula** | **Mass deviation from experimental mass (ppm)** | **Mean intensity (counts)** ± **relative standard deviation** | | | | |
| --- | --- | --- | --- | --- | --- | --- | --- | --- | --- |
|  |  |  |  |  | **Aa (n = 5)** | **Pg (n = 5)** | **Td (n = 5)** | **Tf (n = 5)** | **Medium (n= 5)** |
| Aa | 43.0180 | - | C2H3O | 3.7 | 2393 ± 22 % | 1034 ± 19 % | 1336 ± 10 % | 1498 ± 12 % | 1288 ± 7 % |
| Aa | 58.8706 | - | - | - | 1238 ± 21 % | 584 ± 100 % | 471 ± 27 % | 294 ± 10 % | 232 ± 38 % |
| Aa | 58.9993 | - | - | - | 65 ± 26 % | 30 ± 87 % | 26 ± 29 % | 19 ± 39 % | 15 ± 30 % |
| Aa | 59.0134 | - | C2H3O2 | 11 | 105 ± 12 % | 54 ± 97 % | 41 ± 18 % | 33 ± 22 % | 45 ± 27 % |
| Aa | 59.0481 | 59.0485 | C3H7O | 11 | 141010 ± 20 % | 65786 ± 91 % | 57572 ± 25 % | 38672 ± 11 % | 30688 ± 35 % |
| Aa | 65.0369 | - | C5H5 | 26 | 153 ± 31 % | 59 ± 7 % | 86 ± 8 % | 62 ± 7 % | 57 ± 12 % |
| Aa | 67.0522 | 67.0541 | C5H7 | 1.9 | 1082 ± 31 % | 484 ± 9 % | 543 ± 9 % | 481 ± 4 % | 448 ± 6 % |
| Aa | 84.8031 | - | - | - | 74 ± 46 % | 25 ± 35 % | 40 ± 20 % | 24 ± 14 % | 21 ± 9 % |
| Aa | 85.0630 | 85.0645 | C5H9O | 3.4 | 14851 ± 33 % | 3994 ± 18 % | 6008 ± 11 % | 4055 ± 6 % | 3664 ± 7 % |
| Aa | 87.0667 | - | C2H7N4 | 2 | 49 ± 29 % | 23 ± 12 % | 27 ± 15 % | 25 ± 22 % | 20 ± 14 % |
| Aa | 99.0778 | 99.0793 | C6H11O | 12 | 6316 ± 19 % | 4412 ± 24 % | 3426 ± 7 % | 3779 ± 7 % | 3128 ± 2 % |
| Aa | 117.0886 | 117.0908 | C6H13O2 | 1.8 | 11794 ± 48 % | 4528 ± 9 % | 3223 ± 8 % | 3142 ± 5 % | 2715 ± 4 % |
| Aa | 144.1094 | 144.1097 | C6H14N3O4 | 24 | 213 ± 28 % | 139 ± 3 % | 107 ± 7 % | 107 ± 9 % | 117 ± 7 % |
| Pg | 79.0189 | 79.0177 | C5H3O | 1.8 | 44 ± 20 % | 75 ± 23 % | 52 ± 11 % | 42 ± 19 % | 61 ± 13 % |
| Pg | 88.0738 | 88.0755 | C4H10NO | 2.2 | 1074 ± 5 % | 1665 ± 7 % | 1293 ± 8 % | 1249 ± 4 % | 1418 ± 11 % |
| Pg | 97.0258 | 97.0284 | C5H5O2 | 0.1 | 335 ± 17 % | 935 ± 24 % | 539 ± 11 % | 521 ± 5 % | 697 ± 25 % |
| Pg | 97.0619 | 97.0647 | C6H9O | 0.9 | 1821 ± 15 % | 3156 ± 8 % | 1641 ± 2 % | 1574 ± 8 % | 1784 ± 9 % |
| Pg | 100.0450 | - | C4H6NO2 | 57 | 71 ± 21 % | 145 ± 20 % | 89 ± 18 % | 91 ± 11 % | 114 ± 16 % |
| Pg | 107.0669 | - | C4H11O3 | 31 | 1209 ± 29 % | 1681 ± 21 % | 897 ± 9 % | 994 ± 11 % | 1170 ± 14 % |
| Pg | 110.0579 | - | C6H8NO | 19 | 62 ± 16 % | 98 ± 14 % | 80 ± 9 % | 76 ± 8 % | 85 ± 15 % |
| Pg | 111.0414 | 111.0437 | C6H7O2 | 3.2 | 377 ± 13 % | 844 ± 14 % | 532 ± 7 % | 508 ± 5 % | 551 ± 6 % |
| Pg | 111.0771 | 111.0802 | C7H11O | 0.2 | 1027 ± 13 % | 1790 ± 10 % | 1046 ± 5 % | 965 ± 6 % | 1088 ± 7 % |
| Pg | 113.0573 | 113.0595 | C6H9O2 | 1.8 | 1123 ± 6 % | 2934 ± 20 % | 1472 ± 9 % | 1397 ± 8 % | 1759 ± 13 % |
| Pg | 115.0355 | 115.0363 | C5H7O3 | 23 | 374 ± 16 % | 540 ± 11 % | 407 ± 14 % | 426 ± 5 % | 469 ± 12 % |
| Pg | 115.0730 | 115.0752 | C6H11O2 | 0.2 | 2974 ± 11 % | 4952 ± 10 % | 3323 ± 6 % | 3005 ± 9 % | 3645 ± 12 % |
| Pg | 116.0498 | 116.0484 | C8H6N | 9.3 | 16 ± 25 % | 136 ± 31 % | 43 ± 26 % | 33 ± 27 % | 26 ± 16 % |
| Pg | 116.0680 | 116.0704 | C5H10NO2 | 1.8 | 790 ± 6 % | 1203 ± 5 % | 930 ± 2 % | 881 ± 7 % | 977 ± 6 % |
| Pg | 121.0274 | - | C7H5O2 | 8.3 | 720 ± 11 % | 1017 ± 6 % | 846 ± 7 % | 890 ± 6 % | 924 ± 5 % |
| Pg | 125.0568 | 125.0593 | C7H9O2 | 3.2 | 363 ± 11 % | 527 ± 9 % | 445 ± 7 % | 439 ± 7 % | 483 ± 12 % |
| Pg | 125.0931 | 125.0931 | C8H13O | 24 | 1310 ± 15 % | 1720 ± 7 % | 1104 ± 4 % | 1059 ± 2 % | 1143 ± 7 % |
| Pg | 127.0732 | 127.0751 | C7H11O2 | 2 | 743 ± 5 % | 1762 ± 13 % | 910 ± 13 % | 840 ± 10 % | 1088 ± 11 % |
| Pg | 128.0754 | - | C3H6N5O | 2.2 | 67 ± 7 % | 161 ± 11 % | 88 ± 11 % | 82 ± 8 % | 100 ± 13 % |
| Pg | 128.1042 | 128.1068 | C7H14NO | 0.2 | 832 ± 9 % | 1280 ± 4 % | 1021 ± 8 % | 969 ± 4 % | 1080 ± 6 % |
| Pg | 129.0511 | 129.0518 | C2H5N6O | 1 | 560 ± 10 % | 836 ± 6 % | 640 ± 10 % | 666 ± 9 % | 732 ± 8 % |
| Pg | 129.0881 | 129.0909 | C7H13O2 | 0.8 | 1059 ± 5 % | 1644 ± 7 % | 1233 ± 4 % | 1438 ± 9 % | 1413 ± 6 % |
| Pg | 130.0611 | 130.0647 | C9H8N | 3.3 | 32 ± 16 % | 60 ± 12 % | 49 ± 6 % | 47 ± 14 % | 42 ± 14 % |
| Pg | 130.0838 | 130.0859 | C6H12NO2 | 2.7 | 116 ± 6 % | 192 ± 17 % | 148 ± 9 % | 148 ± 14 % | 166 ± 11 % |
| Pg | 132.0995 | 132.1015 | C6H14NO2 | 3.1 | 179 ± 26 % | 597 ± 19 % | 169 ± 8 % | 209 ± 17 % | 144 ± 10 % |
| Pg | 134.0785 | 134.0808 | C5H12NO3 | 2.8 | 26 ± 20 % | 67 ± 39 % | 40 ± 19 % | 29 ± 16 % | 37 ± 15 % |
| Pg | 136.0722 | 136.0729 | C8H10NO | 21 | 174 ± 7 % | 301 ± 15 % | 239 ± 13 % | 194 ± 9 % | 222 ± 15 % |
| Pg | 137.0216 | 137.0244 | C7H5O3 | 7.9 | 225 ± 8 % | 317 ± 8 % | 245 ± 8 % | 229 ± 10 % | 254 ± 4 % |
| Pg | 138.0143 | - | C6H4NO3 | 31 | 123 ± 8 % | 176 ± 4 % | 135 ± 12 % | 132 ± 14 % | 145 ± 10 % |
| Pg | 140.1120 | 140.1113 | C8H14NO | 31 | 81 ± 16 % | 132 ± 5 % | 101 ± 8 % | 103 ± 7 % | 106 ± 8 % |
| Pg | 141.0990 | - | C8H13O2 | 2.9 | 54 ± 11 % | 86 ± 18 % | 61 ± 5 % | 66 ± 9 % | 68 ± 14 % |
| Pg | 143.1035 | 143.1064 | C8H15O2 | 1.8 | 1485 ± 8 % | 1983 ± 9 % | 1506 ± 3 % | 1501 ± 6 % | 1708 ± 8 % |
| Pg | 144.1349 | 144.1377 | C8H18NO | 4.1 | 103 ± 10 % | 154 ± 9 % | 124 ± 12 % | 124 ± 7 % | 126 ± 6 % |
| Pg | 145.0988 | 145.0969 | C6H13N2O2 | 1.8 | 107 ± 28 % | 201 ± 23 % | 135 ± 10 % | 147 ± 9 % | 169 ± 8 % |
| Pg | 147.0421 | 147.0437 | C9H7O2 | 2.4 | 67 ± 10 % | 106 ± 13 % | 81 ± 20 % | 70 ± 18 % | 80 ± 8 % |
| Pg | 148.0756 | - | C9H10NO | 0.6 | 171 ± 41 % | 1686 ± 60 % | 241 ± 42 % | 324 ± 71 % | 156 ± 43 % |
| Pg | 149.0780 | 149.0805 | C6H13O4 | 2.2 | 39 ± 18 % | 152 ± 39 % | 91 ± 20 % | 81 ± 20 % | 93 ± 25 % |
| Pg | 153.0607 | - | C8H9O3 | 2.1 | 42 ± 16 % | 52 ± 11 % | 38 ± 10 % | 36 ± 13 % | 50 ± 9 % |
| Pg | 155.1042 | 155.1064 | C9H15O2 | 1.7 | 574 ± 9 % | 922 ± 12 % | 731 ± 5 % | 751 ± 5 % | 807 ± 12 % |
| Pg | 157.1191 | 157.1220 | C9H17O2 | 1.9 | 744 ± 7 % | 1142 ± 3 % | 917 ± 6 % | 900 ± 4 % | 991 ± 5 % |
| Pg | 158.0824 | 158.0833 | C7H12NO3 | 13 | 24 ± 21 % | 51 ± 14 % | 34 ± 14 % | 30 ± 23 % | 35 ± 14 % |
| Pg | 158.1515 | 158.1512 | C9H20NO | 17 | 565 ± 7 % | 1243 ± 23 % | 805 ± 15 % | 881 ± 22 % | 786 ± 26 % |
| Pg | 159.1093 | 159.1124 | C7H15N2O2 | 2.5 | 840 ± 7 % | 1236 ± 12 % | 956 ± 8 % | 910 ± 7 % | 991 ± 4 % |
| Pg | 162.0949 | ***-*** | C7H16NOS | 1.2 | 86 ± 14 % | 128 ± 14 % | 92 ± 8 % | 98 ± 12 % | 102 ± 14 % |
| Pg | 163.0716 | 163.0723 | C6H7N6 | 2.3 | 1407 ± 5 % | 1861 ± 9 % | 1556 ± 4 % | 1594 ± 8 % | 1735 ± 5 % |
| Pg | 165.0992 | - | C10H13O2 | 4.3 | 51 ± 9 % | 64 ± 9 % | 53 ± 12 % | 53 ± 9 % | 58 ± 15 % |
| Pg | 171.0982 | 171.0988 | C5H11N6O | 0.5 | 294 ± 8 % | 464 ± 10 % | 379 ± 12 % | 359 ± 10 % | 424 ± 7 % |
| Pg | 171.1351 | 171.1351 | C6H15N6 | 1 | 552 ± 4 % | 851 ± 2 % | 716 ± 3 % | 687 ± 6 % | 774 ± 6 % |
| Pg | 171.1461 | 171.1488 | C9H19N2O | 2.3 | 1823 ± 9 % | 2611 ± 4 % | 2160 ± 5 % | 2053 ± 3 % | 2248 ± 6 % |
| Pg | 172.1664 | 172.1692 | C10H22NO | 2.3 | 75 ± 9 % | 105 ± 15 % | 80 ± 16 % | 78 ± 9 % | 86 ± 5 % |
| Pg | 175.1297 | 175.1300 | C5H15N6O | 1.1 | 190 ± 9 % | 335 ± 19 % | 249 ± 13 % | 221 ± 10 % | 236 ± 10 % |
| Pg | 179.0607 | 179.0631 | C9H11N2S | 3.6 | 402 ± 8 % | 561 ± 8 % | 469 ± 9 % | 454 ± 8 % | 478 ± 6 % |
| Pg | 179.1042 | 179.1038 | C7H11N6 | 1 | 2342 ± 10 % | 3232 ± 5 % | 2750 ± 2 % | 2841 ± 3 % | 3018 ± 8 % |
| Pg | 182.1148 | ***-*** | C10H16NO2 | 15 | 34 ± 21 % | 61 ± 12 % | 47 ± 9 % | 48 ± 7 % | 53 ± 9 % |
| Pg | 185.1506 | 185.1529 | C11H21O2 | 3.8 | 333 ± 12 % | 509 ± 6 % | 413 ± 5 % | 417 ± 4 % | 449 ± 5 % |
| Pg | 189.1455 | 189.1478 | C10H21O3 | 3.8 | 71 ± 32 % | 303 ± 47 % | 161 ± 35 % | 117 ± 15 % | 141 ± 19 % |
| Pg | 191.1394 | 191.1424 | C13H19O | 3.4 | 123 ± 4 % | 207 ± 8 % | 154 ± 9 % | 142 ± 9 % | 158 ± 6 % |
| Pg | 192.1645 | - | C9H22NO3 | 26 | 33 ± 15 % | 58 ± 8 % | 41 ± 8 % | 37 ± 28 % | 38 ± 18 % |
| Pg | 193.1416 | 193.1427 | C9H21O4 | 3.8 | 202 ± 18 % | 416 ± 19 % | 317 ± 16 % | 221 ± 18 % | 269 ± 16 % |
| Pg | 195.1349 | 195.1370 | C12H19O2 | 4.9 | 127 ± 15 % | 198 ± 5 % | 153 ± 17 % | 163 ± 10 % | 173 ± 12 % |
| Pg | 199.1299 | 199.1285 | C7H15N6O | 8.5 | 119 ± 18 % | 181 ± 12 % | 146 ± 5 % | 145 ± 8 % | 149 ± 11 % |
| Pg | 199.1676 | 199.1690 | C12H23O2 | 1.3 | 529 ± 12 % | 879 ± 4 % | 756 ± 9 % | 769 ± 8 % | 862 ± 17 % |
| Pg | 201.1095 | 201.1113 | C10H17O4 | 4.2 | 271 ± 5 % | 362 ± 6 % | 321 ± 2 % | 302 ± 5 % | 333 ± 6 % |
| Pg | 204.1399 | - | C13H18NO | 7.9 | 84 ± 10 % | 774 ± 22 % | 163 ± 8 % | 180 ± 38 % | 108 ± 13 % |
| Pg | 205.1409 | 205.1427 | C10H21O4 | 3.6 | 59 ± 12 % | 211 ± 31 % | 120 ± 38 % | 98 ± 24 % | 99 ± 32 % |
| Pg | 206.1358 | - | C9H20NO4 | 14 | 16 ± 26 % | 45 ± 18 % | 28 ± 20 % | 21 ± 17 % | 22 ± 18 % |
| Pg | 214.0884 | 214.0889 | C10H16NO2S | 3.4 | 757 ± 14 % | 1065 ± 9 % | 900 ± 5 % | 910 ± 4 % | 940 ± 14 % |
| Pg | 235.1863 | - | C15H23O2 | 3.6 | 90 ± 15 % | 150 ± 13 % | 124 ± 11 % | 104 ± 3 % | 107 ± 17 % |
| Pg | 236.1563 | - | C10H22NO5 | 30 | 31 ± 24 % | 84 ± 29 % | 47 ± 18 % | 43 ± 6 % | 49 ± 12 % |
| Pg | 251.1842 | 251.1846 | C12H27O5 | 2.8 | 360 ± 14 % | 647 ± 18 % | 470 ± 15 % | 409 ± 15 % | 436 ± 13 % |
| Td | 119.0570 | - | C7H7N2 | 28 | 18 ± 42 % | 31 ± 22 % | 46 ± 19 % | 29 ± 17 % | 32 ± 14 % |
| Td | 120.0641 | 120.0653 | C4H10NO3 | 1.8 | 14 ± 73 % | 27 ± 59 % | 129 ± 56 % | 40 ± 16 % | 66 ± 53 % |
| Td | 120.0796 | - | C3H10N3O2 | 11 | 136 ± 74 % | 416 ± 29 % | 1710 ± 52 % | 648 ± 45 % | 847 ± 55 % |
| Td | 121.0819 | 121.0855 | C5H13O3 | 3.5 | 31 ± 31 % | 57 ± 21 % | 133 ± 41 % | 73 ± 24 % | 84 ± 34 % |
| Td | 136.1085 | 136.1118 | C9H14N | 2 | 38 ± 27 % | 73 ± 14 % | 104 ± 29 % | 51 ± 9 % | 58 ± 18 % |
| Td | 136.1299 | 136.1329 | - | - | 11 ± 53 % | 19 ± 54 % | 45 ± 39 % | 24 ± 18 % | 28 ± 13 % |
| Td | 150.1237 | 150.1200 | C5H16N3O2 | 25 | 81 ± 9 % | 88 ± 3 % | 104 ± 12 % | 84 ± 9 % | 97 ± 8 % |
| Tf | 44.0492 | - | C2H6N | 6.2 | 64 ± 17 % | 65 ± 21 % | 66 ± 21 % | 111 ± 12 % | 107 ± 22 % |
| Tf | 50.0164 | 50.0152 | - | - | 46 ± 20 % | 51 ± 23 % | 58 ± 13 % | 75 ± 12 % | 69 ± 15 % |
| Tf | 53.0378 | ***-*** | C4H5 | 15 | 28 ± 26 % | 29 ± 10 % | 34 ± 23 % | 52 ± 21 % | 33 ± 23 % |
| Tf | 55.0279 | - | C2H3N2 | 21 | 176 ± 23 % | 89 ± 13 % | 227 ± 7 % | 295 ± 7 % | 271 ± 21 % |
| Tf | 55.0530 | 55.0529 | C4H7 | 24 | 497 ± 37 % | 506 ± 17 % | 841 ± 14 % | 1311 ± 8 % | 672 ± 14 % |
| Tf | 60.0801 | 60.0807 | C3H10N | 1.3 | 150 ± 23 % | 132 ± 37 % | 166 ± 51 % | 433 ± 27 % | 284 ± 33 % |
| Tf | 70.0716 | ***-*** | - | - | 64 ± 32 % | 88 ± 19 % | 98 ± 16 % | 160 ± 4 % | 102 ± 11 % |
| Tf | 73.0441 | 73.0428 | C2H5N2O | 43 | 359 ± 32 % | 374 ± 19 % | 490 ± 21 % | 832 ± 8 % | 528 ± 14 % |
| Tf | 73.0623 | 73.0624 | C4H9O | 33 | 5325 ± 30 % | 5103 ± 21 % | 8835 ± 14 % | 13022 ± 10 % | 5603 ± 39 % |
| Tf | 76.0809 | ***-*** | - | - | 17 ± 17 % | 19 ± 36 % | 42 ± 20 % | 61 ± 16 % | 43 ± 33 % |
| Tf | 77.0362 | ***-*** | C6H5 | 31 | 524 ± 16 % | 634 ± 13 % | 767 ± 10 % | 959 ± 6 % | 804 ± 8 % |
| Tf | 79.0515 | - | C6H7 | 34 | 1437 ± 29 % | 1448 ± 22 % | 2330 ± 13 % | 3222 ± 7 % | 2478 ± 16 % |
| Tf | 87.0785 | 87.0793 | C5H11O | 13 | 810 ± 28 % | 1135 ± 15 % | 1318 ± 13 % | 2203 ± 10 % | 1047 ± 13 % |
| Tf | 89.0577 | 89.0595 | C4H9O2 | 2.3 | 3000 ± 26 % | 3574 ± 23 % | 3387 ± 12 % | 5017 ± 4 % | 4105 ± 9 % |
| Tf | 92.0486 | - | C6H6N | 9.5 | 1060 ± 93 % | 48 ± 53 % | 185 ± 56 % | 5322 ± 32 % | 1623 ± 59 % |
| Tf | 96.0859 | - | C6H10N | 53 | 71 ± 19 % | 97 ± 14 % | 93 ± 6 % | 119 ± 10 % | 103 ± 17 % |
| Tf | 100.0180 | - | C2H3N2O2 | 38 | 57 ± 23 % | 71 ± 34 % | 76 ± 19 % | 124 ± 19 % | 68 ± 22 % |
| Tf | 103.0630 | 103.0601 | C2H7N4O | 13 | 73 ± 30 % | 84 ± 38 % | 123 ± 23 % | 193 ± 15 % | 138 ± 13 % |
| Tf | 103.0729 | 103.0752 | C5H11O2 | 1.5 | 3287 ± 16 % | 2227 ± 18 % | 2144 ± 11 % | 4161 ± 10 % | 2107 ± 2 % |
| Tf | 107.0463 | 107.0436 | C2H7N2O3 | 14 | 5479 ± 34 % | 5651 ± 28 % | 9650 ± 14 % | 13671 ± 7 % | 10360 ± 18 % |
| Tf | 109.0516 | ***-*** | C3H9O4 | 19 | 28 ± 37 % | 31 ± 30 % | 45 ± 17 % | 65 ± 15 % | 44 ± 14 % |
| Tf | 109.0981 | 109.1009 | C8H13 | 2.5 | 967 ± 22 % | 1363 ± 11 % | 1349 ± 7 % | 1719 ± 5 % | 1451 ± 9 % |
| Tf | 115.1081 | 115.1114 | C7H15O | 3 | 655 ± 16 % | 761 ± 8 % | 815 ± 8 % | 1226 ± 7 % | 888 ± 5 % |
| Tf | 116.1120 | ***-*** | C6H14NO | 43 | 63 ± 37 % | 72 ± 9 % | 71 ± 10 % | 107 ± 9 % | 82 ± 9 % |
| Tf | 123.1131 | 123.1166 | C9H15 | 1.8 | 477 ± 21 % | 649 ± 6 % | 641 ± 8 % | 809 ± 6 % | 763 ± 7 % |
| Tf | 127.1082 | 127.1116 | C8H15O | 1.1 | 1019 ± 18 % | 1317 ± 6 % | 1309 ± 7 % | 1577 ± 6 % | 1392 ± 6 % |
| Tf | 142.0290 | - | C9H4NO | 1.8 | 88 ± 47 % | 121 ± 60 % | 69 ± 32 % | 1317 ± 15 % | 250 ± 21 % |
| Tf | 142.0474 | - | C6H8NO3 | 17 | 39 ± 12 % | 49 ± 11 % | 51 ± 8 % | 68 ± 14 % | 54 ± 11 % |
| Tf | 183.1711 | 183.1735 | C12H23O | 4.6 | 377 ± 21 % | 461 ± 9 % | 475 ± 13 % | 680 ± 11 % | 512 ± 17 % |
| Tf | 189.1261 | - | C13H17O | 6.8 | 101 ± 16 % | 136 ± 8 % | 134 ± 18 % | 184 ± 21 % | 109 ± 11 % |

**Table S2.** List with all statistical key values.

| **Bacteria strain specifity** | **m/z measured by QTOF-MS** | **ANOVA / Kruskal - Wallis** | **Storey** | **Multiple Comparison** | | | | |
| --- | --- | --- | --- | --- | --- | --- | --- | --- |
|  |  | **p value** | **q value** | **Compared strains** | **Estimated mean/median difference** | **95 % Confidence interval** | | **Tukey - Kramer** |
|  |  |  |  |  |  | **Lower** | **Upper** | **p values** |
| **Aa** | **43.018** | 1.93E-05 | 1.93E-05 | Aa-Pg | 1.36E+03 | 8.08E+02 | 1.91E+03 | 1.47E-05 |
|  |  |  |  | Aa-Td | 1.06E+03 | 5.06E+02 | 1.61E+03 | 2.62E-04 |
|  |  |  |  | Aa-Tf | 8.94E+02 | 3.44E+02 | 1.45E+03 | 1.38E-03 |
|  |  |  |  | Pg-Td | -3.02E+02 | -8.53E+02 | 2.49E+02 | 4.23E-01 |
|  |  |  |  | Pg-Tf | -4.64E+02 | -1.02E+03 | 8.66E+01 | 1.15E-01 |
|  |  |  |  | Td-Tf | -1.62E+02 | -7.13E+02 | 3.89E+02 | 8.34E-01 |
| **Aa** | **58.8706** | 1.85E-03 | 1.85E-03 | Aa-Pg | 6.54E+02 | 6.16E+01 | 1.25E+03 | 2.80E-02 |
|  |  |  |  | Aa-Td | 7.67E+02 | 1.75E+02 | 1.36E+03 | 9.35E-03 |
|  |  |  |  | Aa-Tf | 9.44E+02 | 3.52E+02 | 1.54E+03 | 1.65E-03 |
|  |  |  |  | Pg-Td | 1.13E+02 | -4.80E+02 | 7.06E+02 | 9.46E-01 |
|  |  |  |  | Pg-Tf | 2.90E+02 | -3.02E+02 | 8.83E+02 | 5.17E-01 |
|  |  |  |  | Td-Tf | 1.77E+02 | -4.16E+02 | 7.70E+02 | 8.28E-01 |
| **Aa** | **58.9993** | 2.04E-03 | 2.04E-03 | Aa-Pg | 3.47E+01 | 4.67E+00 | 6.46E+01 | 2.08E-02 |
|  |  |  |  | Aa-Td | 3.96E+01 | 9.57E+00 | 6.95E+01 | 8.12E-03 |
|  |  |  |  | Aa-Tf | 4.62E+01 | 1.62E+01 | 7.62E+01 | 2.23E-03 |
|  |  |  |  | Pg-Td | 4.89E+00 | -2.51E+01 | 3.49E+01 | 9.65E-01 |
|  |  |  |  | Pg-Tf | 1.16E+01 | -1.84E+01 | 4.15E+01 | 6.93E-01 |
|  |  |  |  | Td-Tf | 6.66E+00 | -2.33E+01 | 3.66E+01 | 9.19E-01 |
| **Aa** | **59.0134** | 3.32E-03 | 3.32E-03 | Aa-Pg | 5.07E+01 | 1.11E+00 | 1.00E+02 | 4.42E-02 |
|  |  |  |  | Aa-Td | 6.38E+01 | 1.43E+01 | 1.13E+02 | 9.74E-03 |
|  |  |  |  | Aa-Tf | 7.19E+01 | 2.23E+01 | 1.21E+02 | 3.76E-03 |
|  |  |  |  | Pg-Td | 1.31E+01 | -3.64E+01 | 6.27E+01 | 8.71E-01 |
|  |  |  |  | Pg-Tf | 2.12E+01 | -2.83E+01 | 7.08E+01 | 6.20E-01 |
|  |  |  |  | Td-Tf | 8.10E+00 | -4.15E+01 | 5.76E+01 | 9.65E-01 |
| **Aa** | **59.0481** | 1.21E-03 | 1.21E-03 | Aa-Pg | 7.52E+04 | 1.34E+04 | 1.37E+05 | 1.47E-02 |
|  |  |  |  | Aa-Td | 8.34E+04 | 2.16E+04 | 1.45E+05 | 6.80E-03 |
|  |  |  |  | Aa-Tf | 1.02E+05 | 4.05E+04 | 1.64E+05 | 1.15E-03 |
|  |  |  |  | Pg-Td | 8.21E+03 | -5.36E+04 | 7.00E+04 | 9.81E-01 |
|  |  |  |  | Pg-Tf | 2.71E+04 | -3.47E+04 | 8.89E+04 | 6.03E-01 |
|  |  |  |  | Td-Tf | 1.89E+04 | -4.29E+04 | 8.07E+04 | 8.18E-01 |
| **Aa** | **65.0369** | 3.35E-05 | 3.35E-05 | Aa-Pg | 9.42E+01 | 5.12E+01 | 1.37E+02 | 6.09E-05 |
|  |  |  |  | Aa-Td | 6.67E+01 | 2.37E+01 | 1.10E+02 | 2.11E-03 |
|  |  |  |  | Aa-Tf | 9.04E+01 | 4.73E+01 | 1.33E+02 | 9.80E-05 |
|  |  |  |  | Pg-Td | -2.75E+01 | -7.05E+01 | 1.56E+01 | 2.97E-01 |
|  |  |  |  | Pg-Tf | -3.87E+00 | -4.69E+01 | 3.92E+01 | 9.94E-01 |
|  |  |  |  | Td-Tf | 2.36E+01 | -1.94E+01 | 6.67E+01 | 4.22E-01 |
| **Aa** | **67.0522** | 8.09E-05 | 8.09E-05 | Aa-Pg | 5.98E+02 | 2.88E+02 | 9.07E+02 | 2.44E-04 |
|  |  |  |  | Aa-Td | 5.39E+02 | 2.29E+02 | 8.48E+02 | 7.12E-04 |
|  |  |  |  | Aa-Tf | 6.01E+02 | 2.91E+02 | 9.10E+02 | 2.32E-04 |
|  |  |  |  | Pg-Td | -5.91E+01 | -3.69E+02 | 2.50E+02 | 9.46E-01 |
|  |  |  |  | Pg-Tf | 2.93E+00 | -3.07E+02 | 3.12E+02 | 1.00E+00 |
|  |  |  |  | Td-Tf | 6.20E+01 | -2.47E+02 | 3.72E+02 | 9.39E-01 |
| **Aa** | **84.8031** | 1.24E-03 | 1.24E-03 | Aa-Pg | 4.98E+01 | 1.72E+01 | 8.24E+01 | 2.43E-03 |
|  |  |  |  | Aa-Td | 3.43E+01 | 1.66E+00 | 6.69E+01 | 3.77E-02 |
|  |  |  |  | Aa-Tf | 5.06E+01 | 1.80E+01 | 8.32E+01 | 2.10E-03 |
|  |  |  |  | Pg-Td | -1.55E+01 | -4.81E+01 | 1.71E+01 | 5.41E-01 |
|  |  |  |  | Pg-Tf | 8.19E-01 | -3.18E+01 | 3.34E+01 | 1.00E+00 |
|  |  |  |  | Td-Tf | 1.63E+01 | -1.63E+01 | 4.89E+01 | 4.99E-01 |
| **Aa** | **85.063** | 8.50E-06 | 8.50E-06 | Aa-Pg | 1.09E+04 | 6.30E+03 | 1.54E+04 | 2.27E-05 |
|  |  |  |  | Aa-Td | 8.84E+03 | 4.28E+03 | 1.34E+04 | 2.34E-04 |
|  |  |  |  | Aa-Tf | 1.08E+04 | 6.24E+03 | 1.54E+04 | 2.43E-05 |
|  |  |  |  | Pg-Td | -2.01E+03 | -6.57E+03 | 2.55E+03 | 5.98E-01 |
|  |  |  |  | Pg-Tf | -6.10E+01 | -4.62E+03 | 4.50E+03 | 1.00E+00 |
|  |  |  |  | Td-Tf | 1.95E+03 | -2.61E+03 | 6.51E+03 | 6.21E-01 |
| **Aa** | **87.0667** | 3.18E-04 | 3.18E-04 | Aa-Pg | 2.62E+01 | 1.16E+01 | 4.08E+01 | 5.25E-04 |
|  |  |  |  | Aa-Td | 2.20E+01 | 7.37E+00 | 3.66E+01 | 2.76E-03 |
|  |  |  |  | Aa-Tf | 2.38E+01 | 9.23E+00 | 3.85E+01 | 1.32E-03 |
|  |  |  |  | Pg-Td | -4.23E+00 | -1.88E+01 | 1.04E+01 | 8.40E-01 |
|  |  |  |  | Pg-Tf | -2.38E+00 | -1.70E+01 | 1.22E+01 | 9.66E-01 |
|  |  |  |  | Td-Tf | 1.85E+00 | -1.28E+01 | 1.65E+01 | 9.83E-01 |
| **Aa** | **99.0778** | 1.92E-04 | 1.92E-04 | Aa-Pg | 1.90E+03 | 4.23E+02 | 3.38E+03 | 9.84E-03 |
|  |  |  |  | Aa-Td | 2.89E+03 | 1.41E+03 | 4.37E+03 | 2.18E-04 |
|  |  |  |  | Aa-Tf | 2.54E+03 | 1.06E+03 | 4.02E+03 | 8.28E-04 |
|  |  |  |  | Pg-Td | 9.86E+02 | -4.94E+02 | 2.47E+03 | 2.64E-01 |
|  |  |  |  | Pg-Tf | 6.33E+02 | -8.48E+02 | 2.11E+03 | 6.22E-01 |
|  |  |  |  | Td-Tf | -3.53E+02 | -1.83E+03 | 1.13E+03 | 9.02E-01 |
| **Aa** | **117.0886** | 4.28E-04 | 4.28E-04 | Aa-Pg | 7.27E+03 | 2.15E+03 | 1.24E+04 | 4.52E-03 |
|  |  |  |  | Aa-Td | 8.57E+03 | 3.45E+03 | 1.37E+04 | 1.03E-03 |
|  |  |  |  | Aa-Tf | 8.65E+03 | 3.53E+03 | 1.38E+04 | 9.44E-04 |
|  |  |  |  | Pg-Td | 1.31E+03 | -3.81E+03 | 6.42E+03 | 8.84E-01 |
|  |  |  |  | Pg-Tf | 1.39E+03 | -3.73E+03 | 6.51E+03 | 8.64E-01 |
|  |  |  |  | Td-Tf | 8.16E+01 | -5.04E+03 | 5.20E+03 | 1.00E+00 |
| **Aa** | **144.1094** | 1.29E-04 | 1.29E-04 | Aa-Pg | 7.47E+01 | 1.92E+01 | 1.30E+02 | 6.99E-03 |
|  |  |  |  | Aa-Td | 1.06E+02 | 5.07E+01 | 1.62E+02 | 2.70E-04 |
|  |  |  |  | Aa-Tf | 1.06E+02 | 5.06E+01 | 1.62E+02 | 2.74E-04 |
|  |  |  |  | Pg-Td | 3.16E+01 | -2.40E+01 | 8.71E+01 | 3.93E-01 |
|  |  |  |  | Pg-Tf | 3.14E+01 | -2.41E+01 | 8.70E+01 | 3.97E-01 |
|  |  |  |  | Td-Tf | -1.47E-01 | -5.57E+01 | 5.54E+01 | 1.00E+00 |
| **Pg** | **79.0189** | 6.18E-04 | 6.18E-04 | Aa-Pg | -3.14E+01 | -5.09E+01 | -1.19E+01 | 1.47E-03 |
|  |  |  |  | Aa-Td | -8.19E+00 | -2.77E+01 | 1.13E+01 | 6.33E-01 |
|  |  |  |  | Aa-Tf | 1.34E+00 | -1.81E+01 | 2.08E+01 | 9.97E-01 |
|  |  |  |  | Pg-Td | 2.32E+01 | 3.74E+00 | 4.27E+01 | 1.69E-02 |
|  |  |  |  | Pg-Tf | 3.27E+01 | 1.33E+01 | 5.22E+01 | 9.91E-04 |
|  |  |  |  | Td-Tf | 9.53E+00 | -9.94E+00 | 2.90E+01 | 5.17E-01 |
| **Pg** | **88.0738** | 7.25E-08 | 7.25E-08 | Aa-Pg | -5.91E+02 | -7.45E+02 | -4.37E+02 | 4.42E-08 |
|  |  |  |  | Aa-Td | -2.19E+02 | -3.73E+02 | -6.56E+01 | 4.35E-03 |
|  |  |  |  | Aa-Tf | -1.75E+02 | -3.29E+02 | -2.14E+01 | 2.29E-02 |
|  |  |  |  | Pg-Td | 3.72E+02 | 2.18E+02 | 5.26E+02 | 1.89E-05 |
|  |  |  |  | Pg-Tf | 4.16E+02 | 2.62E+02 | 5.70E+02 | 4.68E-06 |
|  |  |  |  | Td-Tf | 4.41E+01 | -1.10E+02 | 1.98E+02 | 8.44E-01 |
| **Pg** | **97.0258** | 6.27E-06 | 6.27E-06 | Aa-Pg | -6.00E+02 | -8.17E+02 | -3.82E+02 | 3.62E-06 |
|  |  |  |  | Aa-Td | -2.04E+02 | -4.21E+02 | 1.33E+01 | 6.96E-02 |
|  |  |  |  | Aa-Tf | -1.86E+02 | -4.03E+02 | 3.15E+01 | 1.08E-01 |
|  |  |  |  | Pg-Td | 3.96E+02 | 1.78E+02 | 6.13E+02 | 4.53E-04 |
|  |  |  |  | Pg-Tf | 4.14E+02 | 1.97E+02 | 6.31E+02 | 2.84E-04 |
|  |  |  |  | Td-Tf | 1.82E+01 | -1.99E+02 | 2.36E+02 | 9.95E-01 |
| **Pg** | **97.0619** | 1.48E-09 | 1.48E-09 | Aa-Pg | -1.34E+03 | -1.69E+03 | -9.82E+02 | 5.44E-08 |
|  |  |  |  | Aa-Td | 1.80E+02 | -1.73E+02 | 5.33E+02 | 4.82E-01 |
|  |  |  |  | Aa-Tf | 2.47E+02 | -1.07E+02 | 6.00E+02 | 2.30E-01 |
|  |  |  |  | Pg-Td | 1.52E+03 | 1.16E+03 | 1.87E+03 | 1.22E-08 |
|  |  |  |  | Pg-Tf | 1.58E+03 | 1.23E+03 | 1.94E+03 | 8.36E-09 |
|  |  |  |  | Td-Tf | 6.62E+01 | -2.87E+02 | 4.19E+02 | 9.49E-01 |
| **Pg** | **100.045** | 1.03E-04 | 1.03E-04 | Aa-Pg | -7.35E+01 | -1.08E+02 | -3.89E+01 | 8.69E-05 |
|  |  |  |  | Aa-Td | -1.72E+01 | -5.19E+01 | 1.74E+01 | 5.04E-01 |
|  |  |  |  | Aa-Tf | -1.90E+01 | -5.37E+01 | 1.56E+01 | 4.21E-01 |
|  |  |  |  | Pg-Td | 5.63E+01 | 2.16E+01 | 9.09E+01 | 1.38E-03 |
|  |  |  |  | Pg-Tf | 5.45E+01 | 1.98E+01 | 8.91E+01 | 1.86E-03 |
|  |  |  |  | Td-Tf | -1.80E+00 | -3.65E+01 | 3.28E+01 | 9.99E-01 |
| **Pg** | **107.0669** | 9.38E-04 | 9.38E-04 | Aa-Pg | -4.72E+02 | -9.39E+02 | -4.60E+00 | 4.74E-02 |
|  |  |  |  | Aa-Td | 3.12E+02 | -1.56E+02 | 7.79E+02 | 2.64E-01 |
|  |  |  |  | Aa-Tf | 2.15E+02 | -2.52E+02 | 6.82E+02 | 5.67E-01 |
|  |  |  |  | Pg-Td | 7.84E+02 | 3.16E+02 | 1.25E+03 | 1.02E-03 |
|  |  |  |  | Pg-Tf | 6.87E+02 | 2.20E+02 | 1.15E+03 | 3.38E-03 |
|  |  |  |  | Td-Tf | -9.66E+01 | -5.64E+02 | 3.71E+02 | 9.33E-01 |
| **Pg** | **110.0579** | 3.39E-04 | 3.39E-04 | Aa-Pg | -3.56E+01 | -5.33E+01 | -1.78E+01 | 1.64E-04 |
|  |  |  |  | Aa-Td | -1.72E+01 | -3.50E+01 | 5.39E-01 | 5.90E-02 |
|  |  |  |  | Aa-Tf | -1.38E+01 | -3.15E+01 | 3.96E+00 | 1.59E-01 |
|  |  |  |  | Pg-Td | 1.84E+01 | 6.03E-01 | 3.61E+01 | 4.15E-02 |
|  |  |  |  | Pg-Tf | 2.18E+01 | 4.03E+00 | 3.95E+01 | 1.39E-02 |
|  |  |  |  | Td-Tf | 3.43E+00 | -1.43E+01 | 2.12E+01 | 9.45E-01 |
| **Pg** | **111.0414** | 8.31E-08 | 8.31E-08 | Aa-Pg | -4.67E+02 | -5.90E+02 | -3.43E+02 | 5.54E-08 |
|  |  |  |  | Aa-Td | -1.55E+02 | -2.79E+02 | -3.18E+01 | 1.16E-02 |
|  |  |  |  | Aa-Tf | -1.31E+02 | -2.54E+02 | -7.32E+00 | 3.60E-02 |
|  |  |  |  | Pg-Td | 3.11E+02 | 1.88E+02 | 4.35E+02 | 1.14E-05 |
|  |  |  |  | Pg-Tf | 3.36E+02 | 2.12E+02 | 4.59E+02 | 4.39E-06 |
|  |  |  |  | Td-Tf | 2.45E+01 | -9.91E+01 | 1.48E+02 | 9.40E-01 |
| **Pg** | **111.0771** | 8.71E-09 | 8.71E-09 | Aa-Pg | -7.63E+02 | -9.71E+02 | -5.55E+02 | 8.36E-08 |
|  |  |  |  | Aa-Td | -1.89E+01 | -2.27E+02 | 1.89E+02 | 9.94E-01 |
|  |  |  |  | Aa-Tf | 6.26E+01 | -1.46E+02 | 2.71E+02 | 8.25E-01 |
|  |  |  |  | Pg-Td | 7.44E+02 | 5.36E+02 | 9.52E+02 | 1.17E-07 |
|  |  |  |  | Pg-Tf | 8.25E+02 | 6.17E+02 | 1.03E+03 | 3.00E-08 |
|  |  |  |  | Td-Tf | 8.15E+01 | -1.27E+02 | 2.90E+02 | 6.84E-01 |
| **Pg** | **113.0573** | 3.02E-07 | 3.02E-07 | Aa-Pg | -1.81E+03 | -2.37E+03 | -1.25E+03 | 4.37E-07 |
|  |  |  |  | Aa-Td | -3.49E+02 | -9.08E+02 | 2.11E+02 | 3.16E-01 |
|  |  |  |  | Aa-Tf | -2.74E+02 | -8.33E+02 | 2.85E+02 | 5.16E-01 |
|  |  |  |  | Pg-Td | 1.46E+03 | 9.03E+02 | 2.02E+03 | 7.11E-06 |
|  |  |  |  | Pg-Tf | 1.54E+03 | 9.78E+02 | 2.10E+03 | 3.78E-06 |
|  |  |  |  | Td-Tf | 7.47E+01 | -4.84E+02 | 6.34E+02 | 9.80E-01 |
| **Pg** | **115.0355** | 7.75E-04 | 7.75E-04 | Aa-Pg | -1.66E+02 | -2.61E+02 | -7.11E+01 | 6.75E-04 |
|  |  |  |  | Aa-Td | -3.24E+01 | -1.27E+02 | 6.25E+01 | 7.64E-01 |
|  |  |  |  | Aa-Tf | -5.12E+01 | -1.46E+02 | 4.36E+01 | 4.35E-01 |
|  |  |  |  | Pg-Td | 1.34E+02 | 3.87E+01 | 2.28E+02 | 4.84E-03 |
|  |  |  |  | Pg-Tf | 1.15E+02 | 1.99E+01 | 2.10E+02 | 1.53E-02 |
|  |  |  |  | Td-Tf | -1.88E+01 | -1.14E+02 | 7.60E+01 | 9.40E-01 |
| **Pg** | **115.073** | 2.48E-07 | 2.48E-07 | Aa-Pg | -1.98E+03 | -2.61E+03 | -1.34E+03 | 7.31E-07 |
|  |  |  |  | Aa-Td | -3.49E+02 | -9.84E+02 | 2.85E+02 | 4.19E-01 |
|  |  |  |  | Aa-Tf | -3.08E+01 | -6.65E+02 | 6.04E+02 | 9.99E-01 |
|  |  |  |  | Pg-Td | 1.63E+03 | 9.94E+02 | 2.26E+03 | 9.02E-06 |
|  |  |  |  | Pg-Tf | 1.95E+03 | 1.31E+03 | 2.58E+03 | 9.01E-07 |
|  |  |  |  | Td-Tf | 3.18E+02 | -3.16E+02 | 9.53E+02 | 4.96E-01 |
| **Pg** | **116.0498** | 1.00E-06 | 1.00E-06 | Aa-Pg | -1.21E+02 | -1.61E+02 | -8.01E+01 | 1.34E-06 |
|  |  |  |  | Aa-Td | -2.69E+01 | -6.74E+01 | 1.36E+01 | 2.66E-01 |
|  |  |  |  | Aa-Tf | -1.73E+01 | -5.78E+01 | 2.32E+01 | 6.23E-01 |
|  |  |  |  | Pg-Td | 9.37E+01 | 5.32E+01 | 1.34E+02 | 3.19E-05 |
|  |  |  |  | Pg-Tf | 1.03E+02 | 6.28E+01 | 1.44E+02 | 9.73E-06 |
|  |  |  |  | Td-Tf | 9.64E+00 | -3.08E+01 | 5.01E+01 | 9.03E-01 |
| **Pg** | **116.068** | 3.62E-09 | 3.62E-09 | Aa-Pg | -4.13E+02 | -5.02E+02 | -3.23E+02 | 6.73E-09 |
|  |  |  |  | Aa-Td | -1.40E+02 | -2.29E+02 | -5.06E+01 | 1.92E-03 |
|  |  |  |  | Aa-Tf | -9.14E+01 | -1.81E+02 | -2.18E+00 | 4.37E-02 |
|  |  |  |  | Pg-Td | 2.73E+02 | 1.84E+02 | 3.62E+02 | 9.49E-07 |
|  |  |  |  | Pg-Tf | 3.21E+02 | 2.32E+02 | 4.10E+02 | 1.05E-07 |
|  |  |  |  | Td-Tf | 4.84E+01 | -4.08E+01 | 1.38E+02 | 4.32E-01 |
| **Pg** | **121.0274** | 1.42E-05 | 1.42E-05 | Aa-Pg | -2.97E+02 | -4.09E+02 | -1.84E+02 | 6.49E-06 |
|  |  |  |  | Aa-Td | -1.26E+02 | -2.38E+02 | -1.34E+01 | 2.57E-02 |
|  |  |  |  | Aa-Tf | -1.70E+02 | -2.82E+02 | -5.70E+01 | 2.73E-03 |
|  |  |  |  | Pg-Td | 1.71E+02 | 5.81E+01 | 2.83E+02 | 2.57E-03 |
|  |  |  |  | Pg-Tf | 1.27E+02 | 1.45E+01 | 2.40E+02 | 2.43E-02 |
|  |  |  |  | Td-Tf | -4.36E+01 | -1.56E+02 | 6.89E+01 | 6.90E-01 |
| **Pg** | **125.0568** | 4.59E-05 | 4.59E-05 | Aa-Pg | -1.64E+02 | -2.32E+02 | -9.62E+01 | 1.91E-05 |
|  |  |  |  | Aa-Td | -8.16E+01 | -1.50E+02 | -1.36E+01 | 1.62E-02 |
|  |  |  |  | Aa-Tf | -7.63E+01 | -1.44E+02 | -8.23E+00 | 2.54E-02 |
|  |  |  |  | Pg-Td | 8.27E+01 | 1.46E+01 | 1.51E+02 | 1.48E-02 |
|  |  |  |  | Pg-Tf | 8.80E+01 | 2.00E+01 | 1.56E+02 | 9.41E-03 |
|  |  |  |  | Td-Tf | 5.33E+00 | -6.27E+01 | 7.34E+01 | 9.96E-01 |
| **Pg** | **125.0931** | 4.84E-07 | 4.84E-07 | Aa-Pg | -4.10E+02 | -6.24E+02 | -1.96E+02 | 2.66E-04 |
|  |  |  |  | Aa-Td | 2.06E+02 | -7.84E+00 | 4.20E+02 | 6.10E-02 |
|  |  |  |  | Aa-Tf | 2.51E+02 | 3.70E+01 | 4.65E+02 | 1.89E-02 |
|  |  |  |  | Pg-Td | 6.16E+02 | 4.02E+02 | 8.30E+02 | 2.09E-06 |
|  |  |  |  | Pg-Tf | 6.61E+02 | 4.47E+02 | 8.75E+02 | 8.28E-07 |
|  |  |  |  | Td-Tf | 4.48E+01 | -1.69E+02 | 2.59E+02 | 9.31E-01 |
| **Pg** | **127.0732** | 9.02E-09 | 9.02E-09 | Aa-Pg | -1.02E+03 | -1.27E+03 | -7.67E+02 | 2.32E-08 |
|  |  |  |  | Aa-Td | -1.67E+02 | -4.19E+02 | 8.48E+01 | 2.68E-01 |
|  |  |  |  | Aa-Tf | -9.69E+01 | -3.49E+02 | 1.55E+02 | 6.94E-01 |
|  |  |  |  | Pg-Td | 8.52E+02 | 6.00E+02 | 1.10E+03 | 2.43E-07 |
|  |  |  |  | Pg-Tf | 9.22E+02 | 6.70E+02 | 1.17E+03 | 8.35E-08 |
|  |  |  |  | Td-Tf | 7.00E+01 | -1.82E+02 | 3.22E+02 | 8.55E-01 |
| **Pg** | **128.0754** | 1.30E-09 | 1.30E-09 | Aa-Pg | -9.40E+01 | -1.14E+02 | -7.43E+01 | 5.70E-09 |
|  |  |  |  | Aa-Td | -2.05E+01 | -4.02E+01 | -8.62E-01 | 3.93E-02 |
|  |  |  |  | Aa-Tf | -1.50E+01 | -3.47E+01 | 4.72E+00 | 1.72E-01 |
|  |  |  |  | Pg-Td | 7.34E+01 | 5.37E+01 | 9.31E+01 | 6.56E-08 |
|  |  |  |  | Pg-Tf | 7.90E+01 | 5.93E+01 | 9.87E+01 | 2.57E-08 |
|  |  |  |  | Td-Tf | 5.58E+00 | -1.41E+01 | 2.53E+01 | 8.48E-01 |
| **Pg** | **128.1042** | 1.11E-07 | 1.11E-07 | Aa-Pg | -4.48E+02 | -5.67E+02 | -3.28E+02 | 6.18E-08 |
|  |  |  |  | Aa-Td | -1.89E+02 | -3.09E+02 | -6.96E+01 | 1.76E-03 |
|  |  |  |  | Aa-Tf | -1.36E+02 | -2.56E+02 | -1.69E+01 | 2.26E-02 |
|  |  |  |  | Pg-Td | 2.59E+02 | 1.39E+02 | 3.78E+02 | 6.95E-05 |
|  |  |  |  | Pg-Tf | 3.11E+02 | 1.92E+02 | 4.31E+02 | 7.51E-06 |
|  |  |  |  | Td-Tf | 5.27E+01 | -6.68E+01 | 1.72E+02 | 5.99E-01 |
| **Pg** | **129.0511** | 7.85E-06 | 7.85E-06 | Aa-Pg | -2.76E+02 | -3.78E+02 | -1.74E+02 | 4.56E-06 |
|  |  |  |  | Aa-Td | -7.96E+01 | -1.81E+02 | 2.21E+01 | 1.55E-01 |
|  |  |  |  | Aa-Tf | -1.05E+02 | -2.07E+02 | -3.72E+00 | 4.09E-02 |
|  |  |  |  | Pg-Td | 1.96E+02 | 9.44E+01 | 2.98E+02 | 2.50E-04 |
|  |  |  |  | Pg-Tf | 1.70E+02 | 6.85E+01 | 2.72E+02 | 1.04E-03 |
|  |  |  |  | Td-Tf | -2.59E+01 | -1.28E+02 | 7.59E+01 | 8.85E-01 |
| **Pg** | **129.0881** | 2.28E-07 | 2.28E-07 | Aa-Pg | -5.84E+02 | -7.54E+02 | -4.15E+02 | 1.94E-07 |
|  |  |  |  | Aa-Td | -1.73E+02 | -3.43E+02 | -3.52E+00 | 4.46E-02 |
|  |  |  |  | Aa-Tf | -3.78E+02 | -5.48E+02 | -2.08E+02 | 5.01E-05 |
|  |  |  |  | Pg-Td | 4.11E+02 | 2.41E+02 | 5.81E+02 | 1.87E-05 |
|  |  |  |  | Pg-Tf | 2.06E+02 | 3.63E+01 | 3.76E+02 | 1.50E-02 |
|  |  |  |  | Td-Tf | -2.05E+02 | -3.75E+02 | -3.49E+01 | 1.57E-02 |
| **Pg** | **130.0611** | 6.46E-06 | 6.46E-06 | Aa-Pg | -2.84E+01 | -3.86E+01 | -1.83E+01 | 2.88E-06 |
|  |  |  |  | Aa-Td | -1.77E+01 | -2.78E+01 | -7.56E+00 | 6.85E-04 |
|  |  |  |  | Aa-Tf | -1.55E+01 | -2.57E+01 | -5.40E+00 | 2.34E-03 |
|  |  |  |  | Pg-Td | 1.08E+01 | 6.27E-01 | 2.09E+01 | 3.55E-02 |
|  |  |  |  | Pg-Tf | 1.29E+01 | 2.79E+00 | 2.30E+01 | 1.04E-02 |
|  |  |  |  | Td-Tf | 2.17E+00 | -7.96E+00 | 1.23E+01 | 9.27E-01 |
| **Pg** | **130.0838** | 3.04E-04 | 3.04E-04 | Aa-Pg | -7.54E+01 | -1.13E+02 | -3.83E+01 | 1.43E-04 |
|  |  |  |  | Aa-Td | -3.18E+01 | -6.89E+01 | 5.37E+00 | 1.08E-01 |
|  |  |  |  | Aa-Tf | -3.19E+01 | -6.91E+01 | 5.24E+00 | 1.06E-01 |
|  |  |  |  | Pg-Td | 4.36E+01 | 6.47E+00 | 8.08E+01 | 1.88E-02 |
|  |  |  |  | Pg-Tf | 4.35E+01 | 6.34E+00 | 8.06E+01 | 1.92E-02 |
|  |  |  |  | Td-Tf | -1.36E-01 | -3.73E+01 | 3.70E+01 | 1.00E+00 |
| **Pg** | **132.0995** | 1.61E-08 | 1.61E-08 | Aa-Pg | -4.18E+02 | -5.33E+02 | -3.03E+02 | 9.06E-08 |
|  |  |  |  | Aa-Td | 9.37E+00 | -1.06E+02 | 1.24E+02 | 9.95E-01 |
|  |  |  |  | Aa-Tf | -3.04E+01 | -1.45E+02 | 8.45E+01 | 8.72E-01 |
|  |  |  |  | Pg-Td | 4.28E+02 | 3.13E+02 | 5.42E+02 | 6.74E-08 |
|  |  |  |  | Pg-Tf | 3.88E+02 | 2.73E+02 | 5.03E+02 | 2.51E-07 |
|  |  |  |  | Td-Tf | -3.98E+01 | -1.55E+02 | 7.51E+01 | 7.56E-01 |
| **Pg** | **134.0785** | 9.89E-04 | 9.89E-04 | Aa-Pg | -4.17E+01 | -6.71E+01 | -1.63E+01 | 1.24E-03 |
|  |  |  |  | Aa-Td | -1.47E+01 | -4.01E+01 | 1.07E+01 | 3.75E-01 |
|  |  |  |  | Aa-Tf | -3.68E+00 | -2.91E+01 | 2.17E+01 | 9.75E-01 |
|  |  |  |  | Pg-Td | 2.70E+01 | 1.58E+00 | 5.23E+01 | 3.54E-02 |
|  |  |  |  | Pg-Tf | 3.80E+01 | 1.26E+01 | 6.34E+01 | 2.87E-03 |
|  |  |  |  | Td-Tf | 1.11E+01 | -1.43E+01 | 3.64E+01 | 6.08E-01 |
| **Pg** | **136.0722** | 2.11E-05 | 2.11E-05 | Aa-Pg | -1.27E+02 | -1.81E+02 | -7.35E+01 | 2.35E-05 |
|  |  |  |  | Aa-Td | -6.55E+01 | -1.19E+02 | -1.20E+01 | 1.41E-02 |
|  |  |  |  | Aa-Tf | -2.01E+01 | -7.36E+01 | 3.35E+01 | 7.11E-01 |
|  |  |  |  | Pg-Td | 6.15E+01 | 8.00E+00 | 1.15E+02 | 2.16E-02 |
|  |  |  |  | Pg-Tf | 1.07E+02 | 5.34E+01 | 1.60E+02 | 1.69E-04 |
|  |  |  |  | Td-Tf | 4.54E+01 | -8.07E+00 | 9.90E+01 | 1.11E-01 |
| **Pg** | **137.0216** | 1.13E-05 | 1.13E-05 | Aa-Pg | -9.18E+01 | -1.30E+02 | -5.31E+01 | 2.35E-05 |
|  |  |  |  | Aa-Td | -2.02E+01 | -5.89E+01 | 1.85E+01 | 4.63E-01 |
|  |  |  |  | Aa-Tf | -3.61E+00 | -4.23E+01 | 3.50E+01 | 9.93E-01 |
|  |  |  |  | Pg-Td | 7.16E+01 | 3.29E+01 | 1.10E+02 | 3.81E-04 |
|  |  |  |  | Pg-Tf | 8.81E+01 | 4.95E+01 | 1.27E+02 | 3.79E-05 |
|  |  |  |  | Td-Tf | 1.66E+01 | -2.21E+01 | 5.52E+01 | 6.19E-01 |
| **Pg** | **138.0143** | 6.63E-05 | 6.63E-05 | Aa-Pg | -5.28E+01 | -7.73E+01 | -2.83E+01 | 7.27E-05 |
|  |  |  |  | Aa-Td | -1.27E+01 | -3.72E+01 | 1.18E+01 | 4.72E-01 |
|  |  |  |  | Aa-Tf | -8.85E+00 | -3.34E+01 | 1.57E+01 | 7.33E-01 |
|  |  |  |  | Pg-Td | 4.02E+01 | 1.57E+01 | 6.47E+01 | 1.27E-03 |
|  |  |  |  | Pg-Tf | 4.40E+01 | 1.95E+01 | 6.85E+01 | 5.24E-04 |
|  |  |  |  | Td-Tf | 3.82E+00 | -2.07E+01 | 2.83E+01 | 9.70E-01 |
| **Pg** | **140.112** | 1.70E-06 | 1.70E-06 | Aa-Pg | -5.06E+01 | -6.68E+01 | -3.43E+01 | 7.45E-07 |
|  |  |  |  | Aa-Td | -1.92E+01 | -3.54E+01 | -2.96E+00 | 1.79E-02 |
|  |  |  |  | Aa-Tf | -2.20E+01 | -3.82E+01 | -5.75E+00 | 6.62E-03 |
|  |  |  |  | Pg-Td | 3.14E+01 | 1.51E+01 | 4.76E+01 | 2.45E-04 |
|  |  |  |  | Pg-Tf | 2.86E+01 | 1.23E+01 | 4.48E+01 | 6.40E-04 |
|  |  |  |  | Td-Tf | -2.79E+00 | -1.90E+01 | 1.35E+01 | 9.60E-01 |
| **Pg** | **141.099** | 2.54E-04 | 2.54E-04 | Aa-Pg | -3.21E+01 | -4.84E+01 | -1.58E+01 | 2.01E-04 |
|  |  |  |  | Aa-Td | -6.68E+00 | -2.30E+01 | 9.63E+00 | 6.52E-01 |
|  |  |  |  | Aa-Tf | -1.16E+01 | -2.80E+01 | 4.67E+00 | 2.14E-01 |
|  |  |  |  | Pg-Td | 2.54E+01 | 9.08E+00 | 4.17E+01 | 2.04E-03 |
|  |  |  |  | Pg-Tf | 2.04E+01 | 4.12E+00 | 3.67E+01 | 1.20E-02 |
|  |  |  |  | Td-Tf | -4.96E+00 | -2.13E+01 | 1.13E+01 | 8.20E-01 |
| **Pg** | **143.1035** | 1.18E-05 | 1.18E-05 | Aa-Pg | -4.98E+02 | -7.18E+02 | -2.78E+02 | 4.15E-05 |
|  |  |  |  | Aa-Td | -2.12E+01 | -2.41E+02 | 1.99E+02 | 9.92E-01 |
|  |  |  |  | Aa-Tf | -1.57E+01 | -2.36E+02 | 2.04E+02 | 9.97E-01 |
|  |  |  |  | Pg-Td | 4.77E+02 | 2.57E+02 | 6.97E+02 | 6.86E-05 |
|  |  |  |  | Pg-Tf | 4.82E+02 | 2.62E+02 | 7.02E+02 | 6.01E-05 |
|  |  |  |  | Td-Tf | 5.49E+00 | -2.15E+02 | 2.26E+02 | 1.00E+00 |
| **Pg** | **144.1349** | 7.70E-05 | 7.70E-05 | Aa-Pg | -5.14E+01 | -7.38E+01 | -2.90E+01 | 3.50E-05 |
|  |  |  |  | Aa-Td | -2.16E+01 | -4.40E+01 | 7.42E-01 | 5.99E-02 |
|  |  |  |  | Aa-Tf | -2.11E+01 | -4.35E+01 | 1.30E+00 | 6.85E-02 |
|  |  |  |  | Pg-Td | 2.98E+01 | 7.36E+00 | 5.21E+01 | 7.67E-03 |
|  |  |  |  | Pg-Tf | 3.03E+01 | 7.92E+00 | 5.27E+01 | 6.63E-03 |
|  |  |  |  | Td-Tf | 5.58E-01 | -2.18E+01 | 2.29E+01 | 1.00E+00 |
| **Pg** | **145.0988** | 9.63E-04 | 9.63E-04 | Aa-Pg | -9.47E+01 | -1.48E+02 | -4.14E+01 | 5.73E-04 |
|  |  |  |  | Aa-Td | -2.87E+01 | -8.19E+01 | 2.45E+01 | 4.37E-01 |
|  |  |  |  | Aa-Tf | -4.01E+01 | -9.34E+01 | 1.31E+01 | 1.78E-01 |
|  |  |  |  | Pg-Td | 6.60E+01 | 1.27E+01 | 1.19E+02 | 1.29E-02 |
|  |  |  |  | Pg-Tf | 5.46E+01 | 1.32E+00 | 1.08E+02 | 4.36E-02 |
|  |  |  |  | Td-Tf | -1.14E+01 | -6.46E+01 | 4.18E+01 | 9.26E-01 |
| **Pg** | **147.0421** | 8.27E-04 | 8.27E-04 | Aa-Pg | -3.89E+01 | -6.22E+01 | -1.55E+01 | 1.09E-03 |
|  |  |  |  | Aa-Td | -1.39E+01 | -3.73E+01 | 9.42E+00 | 3.52E-01 |
|  |  |  |  | Aa-Tf | -3.02E+00 | -2.64E+01 | 2.03E+01 | 9.82E-01 |
|  |  |  |  | Pg-Td | 2.49E+01 | 1.59E+00 | 4.83E+01 | 3.42E-02 |
|  |  |  |  | Pg-Tf | 3.58E+01 | 1.25E+01 | 5.92E+01 | 2.30E-03 |
|  |  |  |  | Td-Tf | 1.09E+01 | -1.24E+01 | 3.43E+01 | 5.54E-01 |
| **Pg** | **148.0756** | 7.67E-04 | 7.67E-04 | Aa-Pg | -1.52E+03 | -2.46E+03 | -5.66E+02 | 1.62E-03 |
|  |  |  |  | Aa-Td | -6.97E+01 | -1.02E+03 | 8.79E+02 | 9.97E-01 |
|  |  |  |  | Aa-Tf | -1.53E+02 | -1.10E+03 | 7.95E+02 | 9.66E-01 |
|  |  |  |  | Pg-Td | 1.45E+03 | 4.96E+02 | 2.39E+03 | 2.48E-03 |
|  |  |  |  | Pg-Tf | 1.36E+03 | 4.13E+02 | 2.31E+03 | 4.14E-03 |
|  |  |  |  | Td-Tf | -8.37E+01 | -1.03E+03 | 8.65E+02 | 9.94E-01 |
| **Pg** | **149.078** | 5.36E-04 | 5.36E-04 | Aa-Pg | -1.13E+02 | -1.72E+02 | -5.38E+01 | 2.73E-04 |
|  |  |  |  | Aa-Td | -5.10E+01 | -1.10E+02 | 8.02E+00 | 1.03E-01 |
|  |  |  |  | Aa-Tf | -4.13E+01 | -1.00E+02 | 1.77E+01 | 2.28E-01 |
|  |  |  |  | Pg-Td | 6.18E+01 | 2.77E+00 | 1.21E+02 | 3.86E-02 |
|  |  |  |  | Pg-Tf | 7.15E+01 | 1.25E+01 | 1.31E+02 | 1.52E-02 |
|  |  |  |  | Td-Tf | 9.70E+00 | -4.93E+01 | 6.88E+01 | 9.65E-01 |
| **Pg** | **153.0607** | 1.06E-03 | 1.06E-03 | Aa-Pg | -9.92E+00 | -1.96E+01 | -1.98E-01 | 4.47E-02 |
|  |  |  |  | Aa-Td | 3.96E+00 | -5.76E+00 | 1.37E+01 | 6.56E-01 |
|  |  |  |  | Aa-Tf | 6.32E+00 | -3.40E+00 | 1.60E+01 | 2.83E-01 |
|  |  |  |  | Pg-Td | 1.39E+01 | 4.16E+00 | 2.36E+01 | 4.31E-03 |
|  |  |  |  | Pg-Tf | 1.62E+01 | 6.52E+00 | 2.60E+01 | 1.06E-03 |
|  |  |  |  | Td-Tf | 2.36E+00 | -7.36E+00 | 1.21E+01 | 8.98E-01 |
| **Pg** | **155.1042** | 6.00E-06 | 6.00E-06 | Aa-Pg | -3.48E+02 | -4.71E+02 | -2.26E+02 | 2.41E-06 |
|  |  |  |  | Aa-Td | -1.58E+02 | -2.80E+02 | -3.52E+01 | 9.76E-03 |
|  |  |  |  | Aa-Tf | -1.78E+02 | -3.00E+02 | -5.52E+01 | 3.76E-03 |
|  |  |  |  | Pg-Td | 1.91E+02 | 6.85E+01 | 3.13E+02 | 2.00E-03 |
|  |  |  |  | Pg-Tf | 1.71E+02 | 4.85E+01 | 2.93E+02 | 5.18E-03 |
|  |  |  |  | Td-Tf | -2.00E+01 | -1.42E+02 | 1.02E+02 | 9.65E-01 |
| **Pg** | **157.1191** | 3.70E-09 | 3.70E-09 | Aa-Pg | -3.97E+02 | -4.80E+02 | -3.15E+02 | 5.44E-09 |
|  |  |  |  | Aa-Td | -1.73E+02 | -2.55E+02 | -9.07E+01 | 9.65E-05 |
|  |  |  |  | Aa-Tf | -1.56E+02 | -2.38E+02 | -7.39E+01 | 2.94E-04 |
|  |  |  |  | Pg-Td | 2.24E+02 | 1.42E+02 | 3.06E+02 | 4.28E-06 |
|  |  |  |  | Pg-Tf | 2.41E+02 | 1.59E+02 | 3.23E+02 | 1.68E-06 |
|  |  |  |  | Td-Tf | 1.69E+01 | -6.55E+01 | 9.92E+01 | 9.35E-01 |
| **Pg** | **158.0824** | 2.31E-05 | 2.31E-05 | Aa-Pg | -2.69E+01 | -3.79E+01 | -1.59E+01 | 1.66E-05 |
|  |  |  |  | Aa-Td | -9.49E+00 | -2.05E+01 | 1.51E+00 | 1.04E-01 |
|  |  |  |  | Aa-Tf | -6.33E+00 | -1.73E+01 | 4.67E+00 | 3.82E-01 |
|  |  |  |  | Pg-Td | 1.74E+01 | 6.38E+00 | 2.84E+01 | 1.78E-03 |
|  |  |  |  | Pg-Tf | 2.05E+01 | 9.54E+00 | 3.15E+01 | 3.49E-04 |
|  |  |  |  | Td-Tf | 3.16E+00 | -7.85E+00 | 1.42E+01 | 8.44E-01 |
| **Pg** | **158.1515** | 2.82E-04 | 2.82E-04 | Aa-Pg | -6.78E+02 | -1.01E+03 | -3.44E+02 | 1.44E-04 |
|  |  |  |  | Aa-Td | -2.40E+02 | -5.74E+02 | 9.41E+01 | 2.09E-01 |
|  |  |  |  | Aa-Tf | -3.16E+02 | -6.50E+02 | 1.81E+01 | 6.70E-02 |
|  |  |  |  | Pg-Td | 4.38E+02 | 1.03E+02 | 7.72E+02 | 8.60E-03 |
|  |  |  |  | Pg-Tf | 3.62E+02 | 2.73E+01 | 6.96E+02 | 3.17E-02 |
|  |  |  |  | Td-Tf | -7.60E+01 | -4.10E+02 | 2.58E+02 | 9.14E-01 |
| **Pg** | **159.1093** | 2.63E-05 | 2.63E-05 | Aa-Pg | -3.95E+02 | -5.63E+02 | -2.28E+02 | 2.54E-05 |
|  |  |  |  | Aa-Td | -1.16E+02 | -2.84E+02 | 5.17E+01 | 2.36E-01 |
|  |  |  |  | Aa-Tf | -6.99E+01 | -2.38E+02 | 9.78E+01 | 6.40E-01 |
|  |  |  |  | Pg-Td | 2.79E+02 | 1.12E+02 | 4.47E+02 | 1.08E-03 |
|  |  |  |  | Pg-Tf | 3.26E+02 | 1.58E+02 | 4.93E+02 | 2.31E-04 |
|  |  |  |  | Td-Tf | 4.61E+01 | -1.22E+02 | 2.14E+02 | 8.60E-01 |
| **Pg** | **162.0949** | 3.69E-04 | 3.69E-04 | Aa-Pg | -4.22E+01 | -6.50E+01 | -1.93E+01 | 3.96E-04 |
|  |  |  |  | Aa-Td | -5.91E+00 | -2.88E+01 | 1.70E+01 | 8.80E-01 |
|  |  |  |  | Aa-Tf | -1.26E+01 | -3.55E+01 | 1.02E+01 | 4.16E-01 |
|  |  |  |  | Pg-Td | 3.63E+01 | 1.34E+01 | 5.91E+01 | 1.73E-03 |
|  |  |  |  | Pg-Tf | 2.95E+01 | 6.66E+00 | 5.24E+01 | 9.56E-03 |
|  |  |  |  | Td-Tf | -6.73E+00 | -2.96E+01 | 1.61E+01 | 8.34E-01 |
| **Pg** | **163.0716** | 9.74E-05 | 9.74E-05 | Aa-Pg | -4.54E+02 | -6.59E+02 | -2.50E+02 | 5.18E-05 |
|  |  |  |  | Aa-Td | -1.49E+02 | -3.53E+02 | 5.57E+01 | 2.01E-01 |
|  |  |  |  | Aa-Tf | -1.87E+02 | -3.92E+02 | 1.74E+01 | 7.91E-02 |
|  |  |  |  | Pg-Td | 3.05E+02 | 1.01E+02 | 5.10E+02 | 2.96E-03 |
|  |  |  |  | Pg-Tf | 2.67E+02 | 6.24E+01 | 4.72E+02 | 8.80E-03 |
|  |  |  |  | Td-Tf | -3.83E+01 | -2.43E+02 | 1.66E+02 | 9.49E-01 |
| **Pg** | **165.0992** | 6.82E-03 | 6.82E-03 | Aa-Pg | -1.34E+01 | -2.35E+01 | -3.39E+00 | 7.30E-03 |
|  |  |  |  | Aa-Td | -2.62E+00 | -1.27E+01 | 7.42E+00 | 8.77E-01 |
|  |  |  |  | Aa-Tf | -2.39E+00 | -1.24E+01 | 7.64E+00 | 9.02E-01 |
|  |  |  |  | Pg-Td | 1.08E+01 | 7.72E-01 | 2.08E+01 | 3.26E-02 |
|  |  |  |  | Pg-Tf | 1.10E+01 | 9.92E-01 | 2.11E+01 | 2.88E-02 |
|  |  |  |  | Td-Tf | 2.21E-01 | -9.82E+00 | 1.03E+01 | 1.00E+00 |
| **Pg** | **171.0982** | 5.19E-05 | 5.19E-05 | Aa-Pg | -1.70E+02 | -2.42E+02 | -9.84E+01 | 2.42E-05 |
|  |  |  |  | Aa-Td | -8.48E+01 | -1.57E+02 | -1.29E+01 | 1.82E-02 |
|  |  |  |  | Aa-Tf | -6.51E+01 | -1.37E+02 | 6.86E+00 | 8.35E-02 |
|  |  |  |  | Pg-Td | 8.54E+01 | 1.35E+01 | 1.57E+02 | 1.73E-02 |
|  |  |  |  | Pg-Tf | 1.05E+02 | 3.33E+01 | 1.77E+02 | 3.51E-03 |
|  |  |  |  | Td-Tf | 1.98E+01 | -5.22E+01 | 9.17E+01 | 8.59E-01 |
| **Pg** | **171.1351** | 1.29E-10 | 1.29E-10 | Aa-Pg | -2.99E+02 | -3.48E+02 | -2.49E+02 | 4.03E-09 |
|  |  |  |  | Aa-Td | -1.64E+02 | -2.13E+02 | -1.14E+02 | 3.14E-07 |
|  |  |  |  | Aa-Tf | -1.35E+02 | -1.84E+02 | -8.57E+01 | 3.99E-06 |
|  |  |  |  | Pg-Td | 1.35E+02 | 8.58E+01 | 1.84E+02 | 3.94E-06 |
|  |  |  |  | Pg-Tf | 1.64E+02 | 1.14E+02 | 2.13E+02 | 3.11E-07 |
|  |  |  |  | Td-Tf | 2.87E+01 | -2.06E+01 | 7.79E+01 | 3.74E-01 |
| **Pg** | **171.1461** | 7.37E-08 | 7.37E-08 | Aa-Pg | -7.87E+02 | -9.92E+02 | -5.83E+02 | 4.39E-08 |
|  |  |  |  | Aa-Td | -3.37E+02 | -5.41E+02 | -1.32E+02 | 1.23E-03 |
|  |  |  |  | Aa-Tf | -2.30E+02 | -4.35E+02 | -2.52E+01 | 2.51E-02 |
|  |  |  |  | Pg-Td | 4.51E+02 | 2.46E+02 | 6.56E+02 | 5.71E-05 |
|  |  |  |  | Pg-Tf | 5.57E+02 | 3.53E+02 | 7.62E+02 | 4.31E-06 |
|  |  |  |  | Td-Tf | 1.07E+02 | -9.82E+01 | 3.11E+02 | 4.66E-01 |
| **Pg** | **172.1664** | 2.57E-03 | 2.57E-03 | Aa-Pg | -2.93E+01 | -4.96E+01 | -8.98E+00 | 3.95E-03 |
|  |  |  |  | Aa-Td | -4.16E+00 | -2.44E+01 | 1.61E+01 | 9.35E-01 |
|  |  |  |  | Aa-Tf | -2.69E+00 | -2.30E+01 | 1.76E+01 | 9.81E-01 |
|  |  |  |  | Pg-Td | 2.51E+01 | 4.83E+00 | 4.54E+01 | 1.30E-02 |
|  |  |  |  | Pg-Tf | 2.66E+01 | 6.29E+00 | 4.69E+01 | 8.55E-03 |
|  |  |  |  | Td-Tf | 1.47E+00 | -1.88E+01 | 2.18E+01 | 9.97E-01 |
| **Pg** | **175.1297** | 1.39E-04 | 1.39E-04 | Aa-Pg | -1.45E+02 | -2.15E+02 | -7.55E+01 | 1.06E-04 |
|  |  |  |  | Aa-Td | -5.91E+01 | -1.29E+02 | 1.06E+01 | 1.12E-01 |
|  |  |  |  | Aa-Tf | -3.11E+01 | -1.01E+02 | 3.86E+01 | 5.90E-01 |
|  |  |  |  | Pg-Td | 8.62E+01 | 1.65E+01 | 1.56E+02 | 1.31E-02 |
|  |  |  |  | Pg-Tf | 1.14E+02 | 4.45E+01 | 1.84E+02 | 1.28E-03 |
|  |  |  |  | Td-Tf | 2.80E+01 | -4.17E+01 | 9.77E+01 | 6.66E-01 |
| **Pg** | **179.0607** | 6.86E-05 | 6.86E-05 | Aa-Pg | -1.59E+02 | -2.29E+02 | -8.96E+01 | 3.66E-05 |
|  |  |  |  | Aa-Td | -6.73E+01 | -1.37E+02 | 2.34E+00 | 6.01E-02 |
|  |  |  |  | Aa-Tf | -5.13E+01 | -1.21E+02 | 1.83E+01 | 1.92E-01 |
|  |  |  |  | Pg-Td | 9.19E+01 | 2.23E+01 | 1.62E+02 | 8.04E-03 |
|  |  |  |  | Pg-Tf | 1.08E+02 | 3.82E+01 | 1.77E+02 | 2.12E-03 |
|  |  |  |  | Td-Tf | 1.59E+01 | -5.37E+01 | 8.55E+01 | 9.12E-01 |
| **Pg** | **179.1042** | 1.26E-06 | 1.26E-06 | Aa-Pg | -8.89E+02 | -1.17E+03 | -6.11E+02 | 5.16E-07 |
|  |  |  |  | Aa-Td | -4.08E+02 | -6.86E+02 | -1.30E+02 | 3.41E-03 |
|  |  |  |  | Aa-Tf | -4.98E+02 | -7.76E+02 | -2.20E+02 | 5.29E-04 |
|  |  |  |  | Pg-Td | 4.81E+02 | 2.03E+02 | 7.59E+02 | 7.46E-04 |
|  |  |  |  | Pg-Tf | 3.91E+02 | 1.13E+02 | 6.69E+02 | 4.86E-03 |
|  |  |  |  | Td-Tf | -9.02E+01 | -3.68E+02 | 1.88E+02 | 7.90E-01 |
| **Pg** | **182.1148** | 1.97E-05 | 1.97E-05 | Aa-Pg | -2.68E+01 | -3.71E+01 | -1.64E+01 | 8.09E-06 |
|  |  |  |  | Aa-Td | -1.34E+01 | -2.37E+01 | -3.05E+00 | 9.31E-03 |
|  |  |  |  | Aa-Tf | -1.46E+01 | -2.49E+01 | -4.28E+00 | 4.67E-03 |
|  |  |  |  | Pg-Td | 1.34E+01 | 3.04E+00 | 2.37E+01 | 9.39E-03 |
|  |  |  |  | Pg-Tf | 1.21E+01 | 1.81E+00 | 2.25E+01 | 1.86E-02 |
|  |  |  |  | Td-Tf | -1.22E+00 | -1.16E+01 | 9.11E+00 | 9.86E-01 |
| **Pg** | **185.1506** | 4.34E-07 | 4.34E-07 | Aa-Pg | -1.75E+02 | -2.26E+02 | -1.25E+02 | 1.69E-07 |
|  |  |  |  | Aa-Td | -7.95E+01 | -1.30E+02 | -2.90E+01 | 1.85E-03 |
|  |  |  |  | Aa-Tf | -8.42E+01 | -1.35E+02 | -3.37E+01 | 1.07E-03 |
|  |  |  |  | Pg-Td | 9.60E+01 | 4.55E+01 | 1.47E+02 | 2.87E-04 |
|  |  |  |  | Pg-Tf | 9.12E+01 | 4.07E+01 | 1.42E+02 | 4.87E-04 |
|  |  |  |  | Td-Tf | -4.78E+00 | -5.53E+01 | 4.57E+01 | 9.93E-01 |
| **Pg** | **189.1455** | 1.37E-03 | 1.37E-03 | Aa-Pg | -2.32E+02 | -3.72E+02 | -9.20E+01 | 1.14E-03 |
|  |  |  |  | Aa-Td | -9.00E+01 | -2.30E+02 | 4.98E+01 | 2.91E-01 |
|  |  |  |  | Aa-Tf | -4.52E+01 | -1.85E+02 | 9.46E+01 | 7.92E-01 |
|  |  |  |  | Pg-Td | 1.42E+02 | 1.95E+00 | 2.82E+02 | 4.63E-02 |
|  |  |  |  | Pg-Tf | 1.87E+02 | 4.67E+01 | 3.26E+02 | 7.43E-03 |
|  |  |  |  | Td-Tf | 4.48E+01 | -9.50E+01 | 1.85E+02 | 7.96E-01 |
| **Pg** | **191.1394** | 1.46E-07 | 1.46E-07 | Aa-Pg | -8.37E+01 | -1.07E+02 | -6.04E+01 | 1.12E-07 |
|  |  |  |  | Aa-Td | -3.07E+01 | -5.40E+01 | -7.29E+00 | 8.45E-03 |
|  |  |  |  | Aa-Tf | -1.87E+01 | -4.21E+01 | 4.67E+00 | 1.42E-01 |
|  |  |  |  | Pg-Td | 5.31E+01 | 2.97E+01 | 7.64E+01 | 3.96E-05 |
|  |  |  |  | Pg-Tf | 6.50E+01 | 4.17E+01 | 8.84E+01 | 3.23E-06 |
|  |  |  |  | Td-Tf | 1.20E+01 | -1.14E+01 | 3.53E+01 | 4.80E-01 |
| **Pg** | **192.1645** | 6.97E-05 | 6.97E-05 | Aa-Pg | -2.52E+01 | -3.69E+01 | -1.35E+01 | 7.34E-05 |
|  |  |  |  | Aa-Td | -7.77E+00 | -1.95E+01 | 3.92E+00 | 2.66E-01 |
|  |  |  |  | Aa-Tf | -3.74E+00 | -1.54E+01 | 7.95E+00 | 7.97E-01 |
|  |  |  |  | Pg-Td | 1.74E+01 | 5.72E+00 | 2.91E+01 | 3.02E-03 |
|  |  |  |  | Pg-Tf | 2.14E+01 | 9.74E+00 | 3.31E+01 | 4.21E-04 |
|  |  |  |  | Td-Tf | 4.03E+00 | -7.67E+00 | 1.57E+01 | 7.60E-01 |
| **Pg** | **193.1416** | 3.64E-05 | 3.64E-05 | Aa-Pg | -2.14E+02 | -3.11E+02 | -1.16E+02 | 6.14E-05 |
|  |  |  |  | Aa-Td | -1.15E+02 | -2.13E+02 | -1.72E+01 | 1.86E-02 |
|  |  |  |  | Aa-Tf | -1.92E+01 | -1.17E+02 | 7.85E+01 | 9.42E-01 |
|  |  |  |  | Pg-Td | 9.88E+01 | 1.13E+00 | 1.97E+02 | 4.69E-02 |
|  |  |  |  | Pg-Tf | 1.95E+02 | 9.68E+01 | 2.92E+02 | 1.76E-04 |
|  |  |  |  | Td-Tf | 9.57E+01 | -2.05E+00 | 1.93E+02 | 5.61E-02 |
| **Pg** | **195.1349** | 2.13E-04 | 2.13E-04 | Aa-Pg | -7.03E+01 | -1.04E+02 | -3.65E+01 | 1.09E-04 |
|  |  |  |  | Aa-Td | -2.53E+01 | -5.91E+01 | 8.52E+00 | 1.83E-01 |
|  |  |  |  | Aa-Tf | -3.56E+01 | -6.94E+01 | -1.80E+00 | 3.72E-02 |
|  |  |  |  | Pg-Td | 4.50E+01 | 1.12E+01 | 7.89E+01 | 7.55E-03 |
|  |  |  |  | Pg-Tf | 3.47E+01 | 8.93E-01 | 6.85E+01 | 4.32E-02 |
|  |  |  |  | Td-Tf | -1.03E+01 | -4.41E+01 | 2.35E+01 | 8.18E-01 |
| **Pg** | **199.1299** | 3.30E-04 | 3.30E-04 | Aa-Pg | -6.20E+01 | -9.27E+01 | -3.12E+01 | 1.55E-04 |
|  |  |  |  | Aa-Td | -2.65E+01 | -5.73E+01 | 4.27E+00 | 1.05E-01 |
|  |  |  |  | Aa-Tf | -2.59E+01 | -5.67E+01 | 4.88E+00 | 1.16E-01 |
|  |  |  |  | Pg-Td | 3.55E+01 | 4.70E+00 | 6.62E+01 | 2.12E-02 |
|  |  |  |  | Pg-Tf | 3.61E+01 | 5.31E+00 | 6.68E+01 | 1.89E-02 |
|  |  |  |  | Td-Tf | 6.11E-01 | -3.02E+01 | 3.14E+01 | 1.00E+00 |
| **Pg** | **199.1676** | 8.04E-07 | 8.04E-07 | Aa-Pg | -3.50E+02 | -4.59E+02 | -2.42E+02 | 4.45E-07 |
|  |  |  |  | Aa-Td | -2.27E+02 | -3.35E+02 | -1.19E+02 | 9.88E-05 |
|  |  |  |  | Aa-Tf | -2.40E+02 | -3.48E+02 | -1.31E+02 | 5.34E-05 |
|  |  |  |  | Pg-Td | 1.23E+02 | 1.49E+01 | 2.31E+02 | 2.31E-02 |
|  |  |  |  | Pg-Tf | 1.11E+02 | 2.33E+00 | 2.19E+02 | 4.44E-02 |
|  |  |  |  | Td-Tf | -1.26E+01 | -1.21E+02 | 9.57E+01 | 9.87E-01 |
| **Pg** | **201.1095** | 4.80E-07 | 4.80E-07 | Aa-Pg | -9.08E+01 | -1.18E+02 | -6.39E+01 | 2.49E-07 |
|  |  |  |  | Aa-Td | -5.00E+01 | -7.69E+01 | -2.31E+01 | 3.66E-04 |
|  |  |  |  | Aa-Tf | -3.15E+01 | -5.84E+01 | -4.63E+00 | 1.90E-02 |
|  |  |  |  | Pg-Td | 4.09E+01 | 1.40E+01 | 6.77E+01 | 2.53E-03 |
|  |  |  |  | Pg-Tf | 5.93E+01 | 3.24E+01 | 8.62E+01 | 5.60E-05 |
|  |  |  |  | Td-Tf | 1.84E+01 | -8.45E+00 | 4.53E+01 | 2.43E-01 |
| **Pg** | **204.1399** | 5.05E-09 | 5.05E-09 | Aa-Pg | -6.90E+02 | -8.54E+02 | -5.26E+02 | 1.48E-08 |
|  |  |  |  | Aa-Td | -7.92E+01 | -2.43E+02 | 8.46E+01 | 5.27E-01 |
|  |  |  |  | Aa-Tf | -9.60E+01 | -2.60E+02 | 6.78E+01 | 3.67E-01 |
|  |  |  |  | Pg-Td | 6.11E+02 | 4.47E+02 | 7.74E+02 | 6.58E-08 |
|  |  |  |  | Pg-Tf | 5.94E+02 | 4.30E+02 | 7.58E+02 | 9.55E-08 |
|  |  |  |  | Td-Tf | -1.68E+01 | -1.81E+02 | 1.47E+02 | 9.91E-01 |
| **Pg** | **205.1409** | 2.29E-04 | 2.29E-04 | Aa-Pg | -1.52E+02 | -2.27E+02 | -7.68E+01 | 1.51E-04 |
|  |  |  |  | Aa-Td | -6.13E+01 | -1.37E+02 | 1.41E+01 | 1.33E-01 |
|  |  |  |  | Aa-Tf | -3.94E+01 | -1.15E+02 | 3.59E+01 | 4.62E-01 |
|  |  |  |  | Pg-Td | 9.09E+01 | 1.55E+01 | 1.66E+02 | 1.56E-02 |
|  |  |  |  | Pg-Tf | 1.13E+02 | 3.74E+01 | 1.88E+02 | 2.89E-03 |
|  |  |  |  | Td-Tf | 2.19E+01 | -5.35E+01 | 9.72E+01 | 8.39E-01 |
| **Pg** | **206.1358** | 2.28E-06 | 2.28E-06 | Aa-Pg | -2.89E+01 | -3.91E+01 | -1.88E+01 | 2.36E-06 |
|  |  |  |  | Aa-Td | -1.20E+01 | -2.21E+01 | -1.85E+00 | 1.79E-02 |
|  |  |  |  | Aa-Tf | -4.42E+00 | -1.46E+01 | 5.72E+00 | 6.08E-01 |
|  |  |  |  | Pg-Td | 1.69E+01 | 6.79E+00 | 2.71E+01 | 1.06E-03 |
|  |  |  |  | Pg-Tf | 2.45E+01 | 1.44E+01 | 3.46E+01 | 1.89E-05 |
|  |  |  |  | Td-Tf | 7.57E+00 | -2.57E+00 | 1.77E+01 | 1.84E-01 |
| **Pg** | **214.0884** | 1.90E-04 | 1.90E-04 | Aa-Pg | -3.08E+02 | -4.53E+02 | -1.64E+02 | 8.26E-05 |
|  |  |  |  | Aa-Td | -1.43E+02 | -2.88E+02 | 1.58E+00 | 5.31E-02 |
|  |  |  |  | Aa-Tf | -1.53E+02 | -2.98E+02 | -8.42E+00 | 3.62E-02 |
|  |  |  |  | Pg-Td | 1.65E+02 | 2.05E+01 | 3.10E+02 | 2.25E-02 |
|  |  |  |  | Pg-Tf | 1.55E+02 | 1.05E+01 | 3.00E+02 | 3.33E-02 |
|  |  |  |  | Td-Tf | -1.00E+01 | -1.55E+02 | 1.35E+02 | 9.97E-01 |
| **Pg** | **235.1863** | 2.60E-05 | 2.60E-05 | Aa-Pg | -6.05E+01 | -8.59E+01 | -3.51E+01 | 2.27E-05 |
|  |  |  |  | Aa-Td | -3.48E+01 | -6.02E+01 | -9.37E+00 | 6.07E-03 |
|  |  |  |  | Aa-Tf | -1.42E+01 | -3.96E+01 | 1.12E+01 | 4.08E-01 |
|  |  |  |  | Pg-Td | 2.57E+01 | 3.00E-01 | 5.11E+01 | 4.69E-02 |
|  |  |  |  | Pg-Tf | 4.63E+01 | 2.09E+01 | 7.17E+01 | 4.46E-04 |
|  |  |  |  | Td-Tf | 2.06E+01 | -4.80E+00 | 4.60E+01 | 1.35E-01 |
| **Pg** | **236.1563** | 8.80E-05 | 8.80E-05 | Aa-Pg | -5.21E+01 | -7.63E+01 | -2.80E+01 | 7.01E-05 |
|  |  |  |  | Aa-Td | -1.53E+01 | -3.94E+01 | 8.85E+00 | 3.04E-01 |
|  |  |  |  | Aa-Tf | -1.21E+01 | -3.62E+01 | 1.21E+01 | 4.99E-01 |
|  |  |  |  | Pg-Td | 3.69E+01 | 1.28E+01 | 6.10E+01 | 2.39E-03 |
|  |  |  |  | Pg-Tf | 4.01E+01 | 1.60E+01 | 6.42E+01 | 1.11E-03 |
|  |  |  |  | Td-Tf | 3.20E+00 | -2.09E+01 | 2.73E+01 | 9.81E-01 |
| **Pg** | **251.1842** | 1.55E-04 | 1.55E-04 | Aa-Pg | -2.87E+02 | -4.28E+02 | -1.45E+02 | 1.42E-04 |
|  |  |  |  | Aa-Td | -1.09E+02 | -2.50E+02 | 3.18E+01 | 1.61E-01 |
|  |  |  |  | Aa-Tf | -4.85E+01 | -1.90E+02 | 9.26E+01 | 7.61E-01 |
|  |  |  |  | Pg-Td | 1.77E+02 | 3.61E+01 | 3.18E+02 | 1.17E-02 |
|  |  |  |  | Pg-Tf | 2.38E+02 | 9.69E+01 | 3.79E+02 | 9.65E-04 |
|  |  |  |  | Td-Tf | 6.08E+01 | -8.03E+01 | 2.02E+02 | 6.16E-01 |
| **Td** | **119.057** | 1.84E-04 | 1.84E-04 | Aa-Pg | -1.33E+01 | -2.62E+01 | -4.03E-01 | 4.21E-02 |
|  |  |  |  | Aa-Td | -2.74E+01 | -4.03E+01 | -1.45E+01 | 8.44E-05 |
|  |  |  |  | Aa-Tf | -1.12E+01 | -2.41E+01 | 1.70E+00 | 1.01E-01 |
|  |  |  |  | Pg-Td | -1.41E+01 | -2.70E+01 | -1.24E+00 | 2.93E-02 |
|  |  |  |  | Pg-Tf | 2.10E+00 | -1.08E+01 | 1.50E+01 | 9.66E-01 |
|  |  |  |  | Td-Tf | 1.62E+01 | 3.34E+00 | 2.91E+01 | 1.15E-02 |
| **Td** | **120.0641** | 7.35E-04 | 7.35E-04 | Aa-Pg | -1.30E+01 | -8.09E+01 | 5.49E+01 | 9.46E-01 |
|  |  |  |  | Aa-Td | -1.15E+02 | -1.83E+02 | -4.68E+01 | 9.51E-04 |
|  |  |  |  | Aa-Tf | -2.58E+01 | -9.37E+01 | 4.22E+01 | 7.03E-01 |
|  |  |  |  | Pg-Td | -1.02E+02 | -1.70E+02 | -3.38E+01 | 2.87E-03 |
|  |  |  |  | Pg-Tf | -1.28E+01 | -8.07E+01 | 5.52E+01 | 9.48E-01 |
|  |  |  |  | Td-Tf | 8.90E+01 | 2.10E+01 | 1.57E+02 | 8.58E-03 |
| **Td** | **120.0796** | 5.00E-04 | 5.00E-04 | Aa-Pg | -2.81E+02 | -1.15E+03 | 5.84E+02 | 7.90E-01 |
|  |  |  |  | Aa-Td | -1.57E+03 | -2.44E+03 | -7.09E+02 | 4.54E-04 |
|  |  |  |  | Aa-Tf | -5.12E+02 | -1.38E+03 | 3.53E+02 | 3.59E-01 |
|  |  |  |  | Pg-Td | -1.29E+03 | -2.16E+03 | -4.29E+02 | 2.91E-03 |
|  |  |  |  | Pg-Tf | -2.32E+02 | -1.10E+03 | 6.33E+02 | 8.68E-01 |
|  |  |  |  | Td-Tf | 1.06E+03 | 1.97E+02 | 1.93E+03 | 1.38E-02 |
| **Td** | **121.0819** | 4.33E-04 | 4.33E-04 | Aa-Pg | -2.56E+01 | -7.93E+01 | 2.80E+01 | 5.37E-01 |
|  |  |  |  | Aa-Td | -1.02E+02 | -1.55E+02 | -4.82E+01 | 2.94E-04 |
|  |  |  |  | Aa-Tf | -4.13E+01 | -9.50E+01 | 1.23E+01 | 1.64E-01 |
|  |  |  |  | Pg-Td | -7.62E+01 | -1.30E+02 | -2.25E+01 | 4.51E-03 |
|  |  |  |  | Pg-Tf | -1.57E+01 | -6.93E+01 | 3.79E+01 | 8.36E-01 |
|  |  |  |  | Td-Tf | 6.05E+01 | 6.83E+00 | 1.14E+02 | 2.45E-02 |
| **Td** | **136.1085** | 7.73E-05 | 7.73E-05 | Aa-Pg | -3.50E+01 | -6.55E+01 | -4.53E+00 | 2.17E-02 |
|  |  |  |  | Aa-Td | -6.58E+01 | -9.63E+01 | -3.53E+01 | 7.22E-05 |
|  |  |  |  | Aa-Tf | -1.30E+01 | -4.35E+01 | 1.75E+01 | 6.24E-01 |
|  |  |  |  | Pg-Td | -3.07E+01 | -6.12E+01 | -2.47E-01 | 4.78E-02 |
|  |  |  |  | Pg-Tf | 2.20E+01 | -8.47E+00 | 5.25E+01 | 2.06E-01 |
|  |  |  |  | Td-Tf | 5.28E+01 | 2.23E+01 | 8.33E+01 | 7.52E-04 |
| **Td** | **136.1299** | 1.18E-03 | 1.18E-03 | Aa-Pg | -8.01E+00 | -2.78E+01 | 1.18E+01 | 6.61E-01 |
|  |  |  |  | Aa-Td | -3.39E+01 | -5.37E+01 | -1.40E+01 | 8.50E-04 |
|  |  |  |  | Aa-Tf | -1.32E+01 | -3.30E+01 | 6.60E+00 | 2.64E-01 |
|  |  |  |  | Pg-Td | -2.58E+01 | -4.56E+01 | -6.03E+00 | 8.85E-03 |
|  |  |  |  | Pg-Tf | -5.20E+00 | -2.50E+01 | 1.46E+01 | 8.75E-01 |
|  |  |  |  | Td-Tf | 2.06E+01 | 8.31E-01 | 4.05E+01 | 3.97E-02 |
| **Td** | **150.1237** | 2.55E-03 | 2.55E-03 | Aa-Pg | -7.62E+00 | -2.26E+01 | 7.32E+00 | 4.83E-01 |
|  |  |  |  | Aa-Td | -2.26E+01 | -3.76E+01 | -7.70E+00 | 2.58E-03 |
|  |  |  |  | Aa-Tf | -3.17E+00 | -1.81E+01 | 1.18E+01 | 9.28E-01 |
|  |  |  |  | Pg-Td | -1.50E+01 | -3.00E+01 | -8.76E-02 | 4.84E-02 |
|  |  |  |  | Pg-Tf | 4.44E+00 | -1.05E+01 | 1.94E+01 | 8.29E-01 |
|  |  |  |  | Td-Tf | 1.95E+01 | 4.53E+00 | 3.44E+01 | 8.90E-03 |
| **Tf** | **44.0492** | 6.46E-05 | 6.46E-05 | Aa-Pg | -1.23E+00 | -2.50E+01 | 2.25E+01 | 9.99E-01 |
|  |  |  |  | Aa-Td | -2.21E+00 | -2.59E+01 | 2.15E+01 | 9.93E-01 |
|  |  |  |  | Aa-Tf | -4.66E+01 | -7.03E+01 | -2.29E+01 | 2.04E-04 |
|  |  |  |  | Pg-Td | -9.80E-01 | -2.47E+01 | 2.28E+01 | 9.99E-01 |
|  |  |  |  | Pg-Tf | -4.54E+01 | -6.91E+01 | -2.16E+01 | 2.72E-04 |
|  |  |  |  | Td-Tf | -4.44E+01 | -6.81E+01 | -2.07E+01 | 3.42E-04 |
| **Tf** | **50.0164** | 9.77E-04 | 9.77E-04 | Aa-Pg | -5.39E+00 | -2.24E+01 | 1.17E+01 | 8.03E-01 |
|  |  |  |  | Aa-Td | -1.19E+01 | -2.90E+01 | 5.12E+00 | 2.29E-01 |
|  |  |  |  | Aa-Tf | -2.92E+01 | -4.62E+01 | -1.21E+01 | 8.39E-04 |
|  |  |  |  | Pg-Td | -6.53E+00 | -2.36E+01 | 1.05E+01 | 6.97E-01 |
|  |  |  |  | Pg-Tf | -2.38E+01 | -4.08E+01 | -6.73E+00 | 5.22E-03 |
|  |  |  |  | Td-Tf | -1.72E+01 | -3.43E+01 | -2.00E-01 | 4.69E-02 |
| **Tf** | **53.0378** | 5.14E-04 | 5.14E-04 | Aa-Pg | -1.48E+00 | -1.53E+01 | 1.23E+01 | 9.90E-01 |
|  |  |  |  | Aa-Td | -6.47E+00 | -2.03E+01 | 7.34E+00 | 5.52E-01 |
|  |  |  |  | Aa-Tf | -2.38E+01 | -3.76E+01 | -1.00E+01 | 7.71E-04 |
|  |  |  |  | Pg-Td | -4.99E+00 | -1.88E+01 | 8.83E+00 | 7.33E-01 |
|  |  |  |  | Pg-Tf | -2.24E+01 | -3.62E+01 | -8.54E+00 | 1.43E-03 |
|  |  |  |  | Td-Tf | -1.74E+01 | -3.12E+01 | -3.56E+00 | 1.16E-02 |
| **Tf** | **55.0279** | 4.49E-09 | 4.49E-09 | Aa-Pg | 8.64E+01 | 4.21E+01 | 1.31E+02 | 2.19E-04 |
|  |  |  |  | Aa-Td | -5.15E+01 | -9.59E+01 | -7.24E+00 | 2.00E-02 |
|  |  |  |  | Aa-Tf | -1.20E+02 | -1.64E+02 | -7.53E+01 | 4.81E-06 |
|  |  |  |  | Pg-Td | -1.38E+02 | -1.82E+02 | -9.37E+01 | 7.43E-07 |
|  |  |  |  | Pg-Tf | -2.06E+02 | -2.50E+02 | -1.62E+02 | 6.52E-09 |
|  |  |  |  | Td-Tf | -6.80E+01 | -1.12E+02 | -2.37E+01 | 2.31E-03 |
| **Tf** | **55.053** | 5.54E-08 | 5.54E-08 | Aa-Pg | -9.27E+00 | -2.42E+02 | 2.23E+02 | 9.99E-01 |
|  |  |  |  | Aa-Td | -3.44E+02 | -5.76E+02 | -1.11E+02 | 3.23E-03 |
|  |  |  |  | Aa-Tf | -8.14E+02 | -1.05E+03 | -5.81E+02 | 1.54E-07 |
|  |  |  |  | Pg-Td | -3.34E+02 | -5.67E+02 | -1.02E+02 | 4.07E-03 |
|  |  |  |  | Pg-Tf | -8.04E+02 | -1.04E+03 | -5.72E+02 | 1.80E-07 |
|  |  |  |  | Td-Tf | -4.70E+02 | -7.03E+02 | -2.38E+02 | 1.48E-04 |
| **Tf** | **60.0801** | 3.22E-05 | 3.22E-05 | Aa-Pg | 1.76E+01 | -1.23E+02 | 1.58E+02 | 9.83E-01 |
|  |  |  |  | Aa-Td | -1.57E+01 | -1.56E+02 | 1.25E+02 | 9.88E-01 |
|  |  |  |  | Aa-Tf | -2.83E+02 | -4.24E+02 | -1.43E+02 | 1.51E-04 |
|  |  |  |  | Pg-Td | -3.33E+01 | -1.74E+02 | 1.07E+02 | 9.03E-01 |
|  |  |  |  | Pg-Tf | -3.01E+02 | -4.41E+02 | -1.61E+02 | 7.68E-05 |
|  |  |  |  | Td-Tf | -2.68E+02 | -4.08E+02 | -1.27E+02 | 2.78E-04 |
| **Tf** | **70.0716** | 4.08E-07 | 4.08E-07 | Aa-Pg | -2.46E+01 | -5.32E+01 | 4.11E+00 | 1.07E-01 |
|  |  |  |  | Aa-Td | -3.42E+01 | -6.29E+01 | -5.58E+00 | 1.67E-02 |
|  |  |  |  | Aa-Tf | -9.62E+01 | -1.25E+02 | -6.75E+01 | 2.72E-07 |
|  |  |  |  | Pg-Td | -9.69E+00 | -3.84E+01 | 1.90E+01 | 7.70E-01 |
|  |  |  |  | Pg-Tf | -7.16E+01 | -1.00E+02 | -4.30E+01 | 1.26E-05 |
|  |  |  |  | Td-Tf | -6.19E+01 | -9.06E+01 | -3.33E+01 | 7.06E-05 |
| **Tf** | **73.0441** | 8.28E-07 | 8.28E-07 | Aa-Pg | -1.55E+01 | -1.78E+02 | 1.47E+02 | 9.93E-01 |
|  |  |  |  | Aa-Td | -1.31E+02 | -2.93E+02 | 3.15E+01 | 1.38E-01 |
|  |  |  |  | Aa-Tf | -4.73E+02 | -6.36E+02 | -3.11E+02 | 1.79E-06 |
|  |  |  |  | Pg-Td | -1.15E+02 | -2.78E+02 | 4.70E+01 | 2.17E-01 |
|  |  |  |  | Pg-Tf | -4.58E+02 | -6.20E+02 | -2.95E+02 | 2.77E-06 |
|  |  |  |  | Td-Tf | -3.42E+02 | -5.05E+02 | -1.80E+02 | 9.40E-05 |
| **Tf** | **73.0623** | 1.30E-07 | 1.30E-07 | Aa-Pg | 2.22E+02 | -2.18E+03 | 2.62E+03 | 9.93E-01 |
|  |  |  |  | Aa-Td | -3.51E+03 | -5.91E+03 | -1.11E+03 | 3.49E-03 |
|  |  |  |  | Aa-Tf | -7.70E+03 | -1.01E+04 | -5.30E+03 | 4.93E-07 |
|  |  |  |  | Pg-Td | -3.73E+03 | -6.13E+03 | -1.33E+03 | 2.04E-03 |
|  |  |  |  | Pg-Tf | -7.92E+03 | -1.03E+04 | -5.52E+03 | 3.37E-07 |
|  |  |  |  | Td-Tf | -4.19E+03 | -6.59E+03 | -1.79E+03 | 6.86E-04 |
| **Tf** | **76.0809** | 1.57E-07 | 1.57E-07 | Aa-Pg | -1.28E+00 | -1.49E+01 | 1.23E+01 | 9.93E-01 |
|  |  |  |  | Aa-Td | -2.44E+01 | -3.80E+01 | -1.08E+01 | 5.20E-04 |
|  |  |  |  | Aa-Tf | -4.36E+01 | -5.72E+01 | -3.00E+01 | 5.04E-07 |
|  |  |  |  | Pg-Td | -2.31E+01 | -3.67E+01 | -9.53E+00 | 8.88E-04 |
|  |  |  |  | Pg-Tf | -4.23E+01 | -5.59E+01 | -2.87E+01 | 7.52E-07 |
|  |  |  |  | Td-Tf | -1.92E+01 | -3.28E+01 | -5.57E+00 | 4.79E-03 |
| **Tf** | **77.0362** | 7.41E-07 | 7.41E-07 | Aa-Pg | -1.10E+02 | -2.47E+02 | 2.73E+01 | 1.42E-01 |
|  |  |  |  | Aa-Td | -2.43E+02 | -3.80E+02 | -1.06E+02 | 6.01E-04 |
|  |  |  |  | Aa-Tf | -4.35E+02 | -5.73E+02 | -2.98E+02 | 5.75E-07 |
|  |  |  |  | Pg-Td | -1.33E+02 | -2.70E+02 | 4.27E+00 | 5.93E-02 |
|  |  |  |  | Pg-Tf | -3.26E+02 | -4.63E+02 | -1.88E+02 | 2.36E-05 |
|  |  |  |  | Td-Tf | -1.93E+02 | -3.30E+02 | -5.54E+01 | 4.94E-03 |
| **Tf** | **79.0515** | 2.72E-07 | 2.72E-07 | Aa-Pg | -1.04E+01 | -5.90E+02 | 5.69E+02 | 1.00E+00 |
|  |  |  |  | Aa-Td | -8.93E+02 | -1.47E+03 | -3.14E+02 | 2.23E-03 |
|  |  |  |  | Aa-Tf | -1.78E+03 | -2.36E+03 | -1.21E+03 | 8.58E-07 |
|  |  |  |  | Pg-Td | -8.83E+02 | -1.46E+03 | -3.03E+02 | 2.47E-03 |
|  |  |  |  | Pg-Tf | -1.77E+03 | -2.35E+03 | -1.20E+03 | 9.27E-07 |
|  |  |  |  | Td-Tf | -8.92E+02 | -1.47E+03 | -3.12E+02 | 2.26E-03 |
| **Tf** | **87.0785** | 6.23E-08 | 6.23E-08 | Aa-Pg | -3.26E+02 | -6.91E+02 | 3.92E+01 | 8.89E-02 |
|  |  |  |  | Aa-Td | -5.09E+02 | -8.74E+02 | -1.44E+02 | 5.25E-03 |
|  |  |  |  | Aa-Tf | -1.39E+03 | -1.76E+03 | -1.03E+03 | 4.79E-08 |
|  |  |  |  | Pg-Td | -1.83E+02 | -5.48E+02 | 1.82E+02 | 4.98E-01 |
|  |  |  |  | Pg-Tf | -1.07E+03 | -1.43E+03 | -7.03E+02 | 1.69E-06 |
|  |  |  |  | Td-Tf | -8.85E+02 | -1.25E+03 | -5.20E+02 | 1.81E-05 |
| **Tf** | **89.0577** | 4.73E-04 | 4.73E-04 | Aa-Pg | -5.74E+02 | -1.68E+03 | 5.29E+02 | 4.67E-01 |
|  |  |  |  | Aa-Td | -3.87E+02 | -1.49E+03 | 7.16E+02 | 7.50E-01 |
|  |  |  |  | Aa-Tf | -2.02E+03 | -3.12E+03 | -9.14E+02 | 4.31E-04 |
|  |  |  |  | Pg-Td | 1.87E+02 | -9.16E+02 | 1.29E+03 | 9.61E-01 |
|  |  |  |  | Pg-Tf | -1.44E+03 | -2.55E+03 | -3.41E+02 | 8.61E-03 |
|  |  |  |  | Td-Tf | -1.63E+03 | -2.73E+03 | -5.27E+02 | 3.21E-03 |
| **Tf** | **92.0486** | 6.21E-07 | 6.21E-07 | Aa-Pg | 1.01E+03 | -7.84E+02 | 2.81E+03 | 4.00E-01 |
|  |  |  |  | Aa-Td | 8.75E+02 | -9.21E+02 | 2.67E+03 | 5.21E-01 |
|  |  |  |  | Aa-Tf | -4.26E+03 | -6.06E+03 | -2.47E+03 | 2.36E-05 |
|  |  |  |  | Pg-Td | -1.37E+02 | -1.93E+03 | 1.66E+03 | 9.96E-01 |
|  |  |  |  | Pg-Tf | -5.27E+03 | -7.07E+03 | -3.48E+03 | 1.62E-06 |
|  |  |  |  | Td-Tf | -5.14E+03 | -6.93E+03 | -3.34E+03 | 2.28E-06 |
| **Tf** | **96.0859** | 9.65E-05 | 9.65E-05 | Aa-Pg | -2.56E+01 | -4.67E+01 | -4.60E+00 | 1.45E-02 |
|  |  |  |  | Aa-Td | -2.15E+01 | -4.25E+01 | -4.13E-01 | 4.49E-02 |
|  |  |  |  | Aa-Tf | -4.75E+01 | -6.86E+01 | -2.65E+01 | 4.25E-05 |
|  |  |  |  | Pg-Td | 4.19E+00 | -1.69E+01 | 2.52E+01 | 9.40E-01 |
|  |  |  |  | Pg-Tf | -2.19E+01 | -4.29E+01 | -8.24E-01 | 4.03E-02 |
|  |  |  |  | Td-Tf | -2.61E+01 | -4.71E+01 | -5.01E+00 | 1.30E-02 |
| **Tf** | **100.018** | 3.23E-04 | 3.23E-04 | Aa-Pg | -1.41E+01 | -4.94E+01 | 2.12E+01 | 6.69E-01 |
|  |  |  |  | Aa-Td | -1.91E+01 | -5.44E+01 | 1.62E+01 | 4.33E-01 |
|  |  |  |  | Aa-Tf | -6.73E+01 | -1.03E+02 | -3.20E+01 | 2.79E-04 |
|  |  |  |  | Pg-Td | -5.00E+00 | -4.03E+01 | 3.03E+01 | 9.77E-01 |
|  |  |  |  | Pg-Tf | -5.32E+01 | -8.85E+01 | -1.79E+01 | 2.71E-03 |
|  |  |  |  | Td-Tf | -4.82E+01 | -8.35E+01 | -1.29E+01 | 6.19E-03 |
| **Tf** | **103.063** | 1.75E-05 | 1.75E-05 | Aa-Pg | -1.12E+01 | -6.16E+01 | 3.93E+01 | 9.19E-01 |
|  |  |  |  | Aa-Td | -4.97E+01 | -1.00E+02 | 7.61E-01 | 5.43E-02 |
|  |  |  |  | Aa-Tf | -1.19E+02 | -1.70E+02 | -6.89E+01 | 2.43E-05 |
|  |  |  |  | Pg-Td | -3.85E+01 | -8.89E+01 | 1.19E+01 | 1.70E-01 |
|  |  |  |  | Pg-Tf | -1.08E+02 | -1.59E+02 | -5.78E+01 | 7.68E-05 |
|  |  |  |  | Td-Tf | -6.97E+01 | -1.20E+02 | -1.93E+01 | 5.63E-03 |
| **Tf** | **103.0729** | 1.77E-06 | 1.77E-06 | Aa-Pg | 1.06E+03 | 3.11E+02 | 1.81E+03 | 4.63E-03 |
|  |  |  |  | Aa-Td | 1.14E+03 | 3.94E+02 | 1.89E+03 | 2.42E-03 |
|  |  |  |  | Aa-Tf | -8.74E+02 | -1.62E+03 | -1.26E+02 | 1.95E-02 |
|  |  |  |  | Pg-Td | 8.34E+01 | -6.65E+02 | 8.32E+02 | 9.88E-01 |
|  |  |  |  | Pg-Tf | -1.93E+03 | -2.68E+03 | -1.19E+03 | 8.33E-06 |
|  |  |  |  | Td-Tf | -2.02E+03 | -2.77E+03 | -1.27E+03 | 4.89E-06 |
| **Tf** | **107.0463** | 3.30E-07 | 3.30E-07 | Aa-Pg | -1.72E+02 | -2.85E+03 | 2.51E+03 | 9.98E-01 |
|  |  |  |  | Aa-Td | -4.17E+03 | -6.85E+03 | -1.49E+03 | 2.04E-03 |
|  |  |  |  | Aa-Tf | -8.19E+03 | -1.09E+04 | -5.51E+03 | 9.46E-07 |
|  |  |  |  | Pg-Td | -4.00E+03 | -6.68E+03 | -1.32E+03 | 2.96E-03 |
|  |  |  |  | Pg-Tf | -8.02E+03 | -1.07E+04 | -5.34E+03 | 1.25E-06 |
|  |  |  |  | Td-Tf | -4.02E+03 | -6.70E+03 | -1.34E+03 | 2.81E-03 |
| **Tf** | **109.0516** | 4.33E-05 | 4.33E-05 | Aa-Pg | -2.71E+00 | -1.94E+01 | 1.40E+01 | 9.66E-01 |
|  |  |  |  | Aa-Td | -1.64E+01 | -3.31E+01 | 3.23E-01 | 5.56E-02 |
|  |  |  |  | Aa-Tf | -3.62E+01 | -5.29E+01 | -1.95E+01 | 6.76E-05 |
|  |  |  |  | Pg-Td | -1.37E+01 | -3.04E+01 | 3.03E+00 | 1.30E-01 |
|  |  |  |  | Pg-Tf | -3.35E+01 | -5.02E+01 | -1.68E+01 | 1.61E-04 |
|  |  |  |  | Td-Tf | -1.99E+01 | -3.66E+01 | -3.15E+00 | 1.73E-02 |
| **Tf** | **109.0981** | 5.06E-06 | 5.06E-06 | Aa-Pg | -3.96E+02 | -6.56E+02 | -1.36E+02 | 2.51E-03 |
|  |  |  |  | Aa-Td | -3.82E+02 | -6.42E+02 | -1.21E+02 | 3.44E-03 |
|  |  |  |  | Aa-Tf | -7.52E+02 | -1.01E+03 | -4.92E+02 | 1.99E-06 |
|  |  |  |  | Pg-Td | 1.41E+01 | -2.46E+02 | 2.74E+02 | 9.99E-01 |
|  |  |  |  | Pg-Tf | -3.56E+02 | -6.17E+02 | -9.61E+01 | 6.06E-03 |
|  |  |  |  | Td-Tf | -3.70E+02 | -6.31E+02 | -1.10E+02 | 4.42E-03 |
| **Tf** | **115.1081** | 2.78E-08 | 2.78E-08 | Aa-Pg | -1.06E+02 | -2.51E+02 | 3.87E+01 | 1.96E-01 |
|  |  |  |  | Aa-Td | -1.61E+02 | -3.06E+02 | -1.61E+01 | 2.69E-02 |
|  |  |  |  | Aa-Tf | -5.71E+02 | -7.16E+02 | -4.27E+02 | 3.15E-08 |
|  |  |  |  | Pg-Td | -5.48E+01 | -2.00E+02 | 8.99E+01 | 7.04E-01 |
|  |  |  |  | Pg-Tf | -4.65E+02 | -6.10E+02 | -3.21E+02 | 4.85E-07 |
|  |  |  |  | Td-Tf | -4.11E+02 | -5.55E+02 | -2.66E+02 | 2.54E-06 |
| **Tf** | **116.112** | 4.00E-04 | 4.00E-04 | Aa-Pg | -8.75E+00 | -3.31E+01 | 1.56E+01 | 7.35E-01 |
|  |  |  |  | Aa-Td | -7.73E+00 | -3.20E+01 | 1.66E+01 | 8.00E-01 |
|  |  |  |  | Aa-Tf | -4.42E+01 | -6.85E+01 | -1.99E+01 | 4.57E-04 |
|  |  |  |  | Pg-Td | 1.02E+00 | -2.33E+01 | 2.53E+01 | 9.99E-01 |
|  |  |  |  | Pg-Tf | -3.54E+01 | -5.98E+01 | -1.11E+01 | 3.61E-03 |
|  |  |  |  | Td-Tf | -3.65E+01 | -6.08E+01 | -1.22E+01 | 2.83E-03 |
| **Tf** | **123.1131** | 5.57E-06 | 5.57E-06 | Aa-Pg | -1.71E+02 | -2.87E+02 | -5.58E+01 | 3.13E-03 |
|  |  |  |  | Aa-Td | -1.63E+02 | -2.79E+02 | -4.77E+01 | 4.70E-03 |
|  |  |  |  | Aa-Tf | -3.31E+02 | -4.47E+02 | -2.16E+02 | 2.19E-06 |
|  |  |  |  | Pg-Td | 8.10E+00 | -1.07E+02 | 1.24E+02 | 9.97E-01 |
|  |  |  |  | Pg-Tf | -1.60E+02 | -2.76E+02 | -4.46E+01 | 5.50E-03 |
|  |  |  |  | Td-Tf | -1.68E+02 | -2.84E+02 | -5.27E+01 | 3.65E-03 |
| **Tf** | **127.1082** | 1.70E-05 | 1.70E-05 | Aa-Pg | -2.98E+02 | -5.11E+02 | -8.54E+01 | 5.04E-03 |
|  |  |  |  | Aa-Td | -2.90E+02 | -5.03E+02 | -7.74E+01 | 6.26E-03 |
|  |  |  |  | Aa-Tf | -5.59E+02 | -7.72E+02 | -3.46E+02 | 6.87E-06 |
|  |  |  |  | Pg-Td | 7.92E+00 | -2.05E+02 | 2.21E+02 | 1.00E+00 |
|  |  |  |  | Pg-Tf | -2.60E+02 | -4.73E+02 | -4.74E+01 | 1.42E-02 |
|  |  |  |  | Td-Tf | -2.68E+02 | -4.81E+02 | -5.53E+01 | 1.15E-02 |
| **Tf** | **142.029** | 4.13E-12 | 4.13E-12 | Aa-Pg | -3.36E+01 | -2.30E+02 | 1.63E+02 | 9.61E-01 |
|  |  |  |  | Aa-Td | 1.82E+01 | -1.79E+02 | 2.15E+02 | 9.93E-01 |
|  |  |  |  | Aa-Tf | -1.23E+03 | -1.43E+03 | -1.03E+03 | 4.02E-09 |
|  |  |  |  | Pg-Td | 5.17E+01 | -1.45E+02 | 2.48E+02 | 8.74E-01 |
|  |  |  |  | Pg-Tf | -1.20E+03 | -1.39E+03 | -9.99E+02 | 4.03E-09 |
|  |  |  |  | Td-Tf | -1.25E+03 | -1.44E+03 | -1.05E+03 | 4.01E-09 |
| **Tf** | **142.0474** | 2.52E-05 | 2.52E-05 | Aa-Pg | -9.92E+00 | -2.15E+01 | 1.64E+00 | 1.06E-01 |
|  |  |  |  | Aa-Td | -1.20E+01 | -2.36E+01 | -4.50E-01 | 4.03E-02 |
|  |  |  |  | Aa-Tf | -2.89E+01 | -4.05E+01 | -1.73E+01 | 1.25E-05 |
|  |  |  |  | Pg-Td | -2.09E+00 | -1.36E+01 | 9.47E+00 | 9.54E-01 |
|  |  |  |  | Pg-Tf | -1.90E+01 | -3.05E+01 | -7.41E+00 | 1.25E-03 |
|  |  |  |  | Td-Tf | -1.69E+01 | -2.84E+01 | -5.33E+00 | 3.56E-03 |
| **Tf** | **183.1711** | 1.65E-05 | 1.65E-05 | Aa-Pg | -8.47E+01 | -2.05E+02 | 3.52E+01 | 2.21E-01 |
|  |  |  |  | Aa-Td | -9.89E+01 | -2.19E+02 | 2.11E+01 | 1.26E-01 |
|  |  |  |  | Aa-Tf | -3.04E+02 | -4.24E+02 | -1.84E+02 | 1.07E-05 |
|  |  |  |  | Pg-Td | -1.41E+01 | -1.34E+02 | 1.06E+02 | 9.86E-01 |
|  |  |  |  | Pg-Tf | -2.19E+02 | -3.39E+02 | -9.89E+01 | 4.41E-04 |
|  |  |  |  | Td-Tf | -2.05E+02 | -3.25E+02 | -8.48E+01 | 8.59E-04 |
| **Tf** | **189.1261** | 7.80E-04 | 7.80E-04 | Aa-Pg | -3.59E+01 | -8.12E+01 | 9.37E+00 | 1.47E-01 |
|  |  |  |  | Aa-Td | -3.38E+01 | -7.91E+01 | 1.15E+01 | 1.84E-01 |
|  |  |  |  | Aa-Tf | -8.38E+01 | -1.29E+02 | -3.85E+01 | 3.84E-04 |
|  |  |  |  | Pg-Td | 2.11E+00 | -4.32E+01 | 4.74E+01 | 9.99E-01 |
|  |  |  |  | Pg-Tf | -4.79E+01 | -9.31E+01 | -2.56E+00 | 3.66E-02 |
|  |  |  |  | Td-Tf | -5.00E+01 | -9.52E+01 | -4.67E+00 | 2.82E-02 |

**Table S3.** Results of the saliva targeted analysis of the 120 compounds found to distinguish the *in vitro* cultures. Compounds in bold were found at least a factor of two more intense in the saliva of the patient vs. two controls.

| **Bacteria specificity** | **Bacteria cultures** | **Saliva** | **Present in patient’s saliva (IAI Pado-Test 4.5)** | **Enhanced in patient vs. controls** | **Enhancement ratio** | | **Mean intensity (counts)** ± **relative standard deviation** | | |
| --- | --- | --- | --- | --- | --- | --- | --- | --- | --- |
|  | **m/z** | **m/z** |  |  | **patient / control 1** | **patient / control 2** | **patient 1 (n = 3)** | **control 1 (n = 3)** | **control 2 (n = 3)** |
| Aa | 43.0180 | - | no | not present | - | - | - | - | - |
| Aa | 58.8706 | 58.8693 | no | no | 0.18 | 0.15 | 14 + 9 % | 74 + 17 % | 89 + 38 % |
| Aa | 58.9993 | - | no | not present | - | - | - | - | - |
| Aa | 59.0134 | - | no | not present | - | - | - | - | - |
| Aa | 59.0481 | 59.0490 | no | no | 0.22 | 0.15 | 1957 + 10 % | 9019 + 20 % | 12743 + 44 % |
| Aa | 65.0369 | - | no | yes | 27.42 | 88.89 | 1090 + 15 % | 40 + 39 % | 12 + 37 % |
| Aa | 67.0522 | 67.0535 | no | no | 0.43 | 0.31 | 49 + 8 % | 116 + 3 % | 158 + 23 % |
| Aa | 84.8031 | - | no | not present | - | - | - | - | - |
| Aa | 85.0630 | 85.0630 | no | no | 0.50 | 0.42 | 261 + 13 % | 520 + 2 % | 615 + 11 % |
| Aa | 87.0667 | - | no | not present | - | - | - | - | - |
| Aa | 99.0778 | 99.0790 | no | no | 0.33 | 0.18 | 397 + 26 % | 1217 + 9 % | 2179 + 53 % |
| Aa | 117.0886 | 117.0887 | no | no | 1.23 | 1.33 | 1110 + 45 % | 900 + 2 % | 833 + 13 % |
| Aa | 144.1094 | 144.1074 | no | no | 0.82 | 0.86 | 174 + 24 % | 211 + 14 % | 202 + 34 % |
| **Pg** | **79.0189** | **79.0198** | **yes** | **yes** | **3.28** | **4.65** | **88 + 37 %** | **27 + 11 %** | **19 + 46 %** |
| Pg | 88.0738 | 88.0737 | yes | no | 1.55 | 2.21 | 1038 + 24 % | 669 + 21 % | 469 + 35 % |
| Pg | 97.0258 | 97.0267 | yes | no | 0.34 | 0.30 | 79 + 22 % | 230 + 6 % | 260 + 21 % |
| Pg | 97.0619 | 97.0630 | yes | no | 0.47 | 0.48 | 390 + 9 % | 836 + 3 % | 820 + 4 % |
| Pg | 100.0450 | 100.0464 | yes | no | 1.15 | 1.23 | 45 + 25 % | 39 + 13 % | 36 + 32 % |
| **Pg** | **107.0669** | **107.0683** | **yes** | **yes** | **3.06** | **3.53** | **1424 + 12 %** | **465 + 18 %** | **404 + 47 %** |
| Pg | 110.0579 | 110.0579 | yes | no | 1.13 | 1.40 | 55 + 31 % | 49 + 17 % | 40 + 18 % |
| Pg | 111.0414 | 111.0419 | yes | no | 0.37 | 0.38 | 123 + 15 % | 336 + 3 % | 322 + 14 % |
| Pg | 111.0771 | 111.0785 | yes | no | 1.02 | 1.12 | 1873 + 31 % | 1842 + 8 % | 1668 + 39 % |
| Pg | 113.0573 | 113.0574 | yes | no | 0.94 | 1.38 | 1707 + 55 % | 1812 + 3 % | 1233 + 5 % |
| Pg | 115.0355 | 115.0362 | yes | no | 0.20 | 0.23 | 69 + 17 % | 337 + 3 % | 304 + 14 % |
| Pg | 115.0730 | 115.0727 | yes | no | 0.75 | 0.85 | 1128 + 31 % | 1507 + 5 % | 1334 + 16 % |
| Pg | 116.0498 | 116.0492 | yes | no | 0.62 | 0.44 | 58 + 13 % | 94 + 72 % | 130 + 110 % |
| Pg | 116.0680 | 116.0691 | yes | no | 0.84 | 1.08 | 301 + 16 % | 359 + 17 % | 279 + 26 % |
| Pg | 121.0274 | 121.0291 | yes | no | 1.19 | 1.26 | 212 + 46 % | 179 + 10 % | 169 + 29 % |
| Pg | 125.0568 | 125.0569 | yes | no | 0.33 | 0.40 | 119 + 18 % | 362 + 4 % | 300 + 10 % |
| Pg | 125.0931 | 125.0933 | yes | no | 1.37 | 1.55 | 1716 + 42 % | 1250 + 11 % | 1109 + 23 % |
| Pg | 127.0732 | 127.0734 | yes | no | 0.60 | 0.81 | 545 + 27 % | 904 + 7 % | 676 + 10 % |
| Pg | 128.0754 | 128.0759 | yes | no | 0.67 | 0.84 | 55 + 24 % | 82 + 5 % | 66 + 18 % |
| **Pg** | **128.1042** | 128.1044 | **yes** | **yes** | **2.41** | **3.03** | **438 + 13 %** | **181 + 12 %** | **144 + 16 %** |
| Pg | 129.0511 | 129.0530 | yes | no | 0.68 | 0.68 | 225 + 23 % | 332 + 2 % | 333 + 20 % |
| Pg | 129.0881 | 129.0898 | yes | no | 0.77 | 0.83 | 4858 + 36 % | 6314 + 21 % | 5842 + 67 % |
| **Pg** | **130.0611** | **130.0645** | **yes** | **yes** | **2.50** | **2.67** | **63 + 28 %** | **25 + 20 %** | **24 + 28 %** |
| Pg | 130.0838 | 130.0838 | yes | no | 1.21 | 1.68 | 162 + 26 % | 134 + 13 % | 96 + 18 % |
| Pg | 132.0995 | 132.0995 | yes | no | 1.34 | 1.73 | 118 + 20 % | 88 + 9 % | 68 + 47 % |
| Pg | 134.0785 | - | yes | not present | **-** | **-** | **-** | **-** | **-** |
| Pg | 136.0722 | 136.0731 | yes | no | 0.93 | 1.32 | 95 + 15 % | 102 + 14 % | 72 + 22 % |
| **Pg** | **137.0216** | 137.0220 | **yes** | **yes** | **2.35** | **2.75** | **253 + 22 %** | **108 + 11 %** | **92 + 7 %** |
| **Pg** | **138.0143** | 138.0146 | **yes** | **yes** | **2.49** | **2.89** | **133 + 21 %** | **53 + 14 %** | **46 + 9 %** |
| Pg | 140.1120 | 140.1148 | yes | no | 0.55 | 0.64 | 122 + 29 % | 223 + 6 % | 192 + 48 % |
| Pg | 141.0990 | - | yes | not present | - | - | - | - | - |
| Pg | 143.1035 | 143.1048 | yes | no | 0.87 | 0.80 | 1838 + 22 % | 2111 + 13 % | 2288 + 37 % |
| Pg | 144.1349 | 144.1350 | yes | no | 1.75 | 2.51 | 150 + 26 % | 86 + 19 % | 60 + 22 % |
| Pg | 145.0988 | 145.0984 | yes | no | 0.41 | 0.43 | 45 + 11 % | 111 + 10 % | 107 + 7 % |
| Pg | 147.0421 | 147.0422 | yes | no | 0.27 | 0.31 | 19 + 24 % | 71 + 4 % | 62 + 11 % |
| **Pg** | **148.0756** | **148.0756** | **yes** | **yes** | **4.07** | **4.39** | **68 + 5 %** | **17 + 20 %** | **15 + 26 %** |
| Pg | 149.0780 | 149.0766 | yes | no | 1.27 | 2.02 | 57 + 27 % | 44 + 7 % | 28 + 35 % |
| Pg | 153.0607 | - | yes | not present | - | - | - | - | - |
| Pg | 155.1042 | 155.1049 | yes | no | 0.60 | 0.92 | 468 + 14 % | 783 + 6 % | 508 + 5 % |
| Pg | 157.1191 | 157.1198 | yes | no | 0.56 | 0.65 | 675 + 17 % | 1198 + 10 % | 1035 + 26 % |
| Pg | 158.0824 | - | yes | not present | - | - | - | - | - |
| **Pg** | **158.1515** | 158.1518 | **yes** | **yes** | **7.06** | **8.68** | **3357 + 21 %** | **475 + 11 %** | **387 + 18 %** |
| **Pg** | **159.1093** | 159.1114 | **yes** | **yes** | **2.04** | **2.96** | **248 + 30 %** | **122 + 1 %** | **84 + 9 %** |
| Pg | 162.0949 | - | yes | not present | - | - | - | - | - |
| Pg | 163.0716 | 163.0729 | yes | no | 0.63 | 0.76 | 120 + 19 % | 191 + 4 % | 159 + 15 % |
| **Pg** | **165.0992** | 165.0995 | **yes** | **yes** | **2.06** | **2.76** | **112 + 53 %** | **54 + 1 %** | **41 + 6 %** |
| Pg | 171.0982 | 171.0986 | yes | no | 0.94 | 1.33 | 397 + 42 % | 422 + 6 % | 298 + 8 % |
| Pg | 171.1351 | 171.1352 | yes | no | 0.64 | 0.79 | 435 + 16 % | 676 + 9 % | 551 + 14 % |
| Pg | 171.1461 | 171.1465 | yes | no | 1.91 | 2.42 | 1012 + 26 % | 530 + 11 % | 418 + 6 % |
| Pg | 172.1664 | 172.1668 | yes | no | 1.65 | 1.82 | 110 + 18 % | 67 + 10 % | 61 + 23 % |
| Pg | 175.1297 | 175.1301 | yes | no | 1.98 | 2.21 | 256 + 42 % | 129 + 10 % | 116 + 43 % |
| Pg | 179.0607 | 179.0613 | yes | no | 1.06 | 1.69 | 251 + 22 % | 235 + 20 % | 148 + 12 % |
| Pg | 179.1042 | 179.1047 | yes | no | 0.48 | 0.59 | 237 + 10 % | 497 + 7 % | 405 + 12 % |
| Pg | 182.1148 | - | yes | not present | - | - | - | - | - |
| Pg | 185.1506 | 185.1515 | yes | no | 0.47 | 0.53 | 221 + 4 % | 468 + 14 % | 413 + 7 % |
| **Pg** | **189.1455** | 189.1459 | **yes** | **yes** | **2.34** | **2.52** | **232 + 75 %** | **99 + 6 %** | **92 + 42 %** |
| Pg | 191.1394 | 191.1406 | yes | no | 0.67 | 0.88 | 105 + 13 % | 156 + 14 % | 118 + 15 % |
| Pg | 192.1645 | - | yes | not present | - | - | - | - | - |
| Pg | 193.1416 | 193.1428 | yes | no | 3.10 | 0.91 | 163 + 41 % | 52 + 13 % | 179 + 64 % |
| Pg | 195.1349 | 195.1366 | yes | no | 0.60 | 0.67 | 80 + 13 % | 133 + 14 % | 119 + 8 % |
| Pg | 199.1299 | 199.1299 | yes | no | 0.62 | 0.88 | 117 + 22 % | 189 + 9 % | 134 + 19 % |
| Pg | 199.1676 | 199.1681 | yes | no | 1.15 | 1.28 | 847 + 8 % | 738 + 7 % | 663 + 16 % |
| Pg | 201.1095 | 201.1102 | yes | no | 1.59 | 2.37 | 234 + 35 % | 147 + 3 % | 98 + 9 % |
| **Pg** | **204.1399** | 204.1403 | **yes** | **yes** | **3.35** | **4.18** | **49 + 5 %** | **15 + 11 %** | **12 + 12 %** |
| Pg | 205.1409 | 205.1413 | yes | no | 1.30 | 2.33 | 113 + 41 % | 86 + 7 % | 48 + 36 % |
| Pg | 206.1358 | - | yes | not present | - | - | - | - | - |
| Pg | 214.0884 | 214.0873 | yes | no | 0.18 | 0.22 | 80 + 10 % | 452 + 12 % | 358 + 17 % |
| Pg | 235.1863 | - | yes | not present | - | - | - | - | - |
| Pg | 236.1563 | - | yes | not present | - | - | - | - | - |
| **Pg** | **251.1842** | 251.1849 | **yes** | **yes** | **7.77** | **7.74** | **127 + 32 %** | **16 + 12 %** | **16 + 32 %** |
| Td | 119.0570 | - | yes | - | - | - | - | - | - |
| Td | 120.0641 | 120.0638 | yes | no | 0.02 | 0.03 | 11 + 13 % | 551 + 24 % | 311 + 61 % |
| Td | 120.0796 | 120.0807 | yes | no | 0.67 | 0.41 | 17 + 32 % | 26 + 16 % | 42 + 38 % |
| **Td** | **121.0819** | 121.0835 | **yes** | **yes** | **2.47** | **2.36** | **95 + 25 %** | **38 + 25 %** | **40 + 56 %** |
| Td | 136.1085 | 136.1094 | yes | no | 0.53 | 1.09 | 92 + 22 % | 174 + 28 % | 85 + 49 % |
| Td | 136.1299 | 136.1307 | yes | no | 0.02 | 0.03 | 5 + 88 % | 310 + 15 % | 201 + 59 % |
| Td | 150.1237 | 150.1253 | yes | no | 0.57 | 0.60 | 43 + 43 % | 75 + 23 % | 72 + 63 % |
| Tf | 44.0492 | - | yes | not present | - | - | - | - | - |
| Tf | 50.0164 | - | yes | not present | - | - | - | - | - |
| Tf | 53.0378 | - | yes | not present | - | - | - | - | - |
| Tf | 55.0279 | - | yes | not present | - | - | - | - | - |
| Tf | 55.0530 | 55.0534 | yes | no | 0.65 | 0.52 | 186 + 13 % | 288 + 4 % | 359 + 17 % |
| Tf | 60.0801 | 60.0800 | yes | no | 0.55 | 0.92 | 102 + 26 % | 185 + 25 % | 111 + 58 % |
| Tf | 70.0716 | - | yes | not present | - | - | - | - | - |
| Tf | 73.0441 | 73.0458 | yes | no | 0.22 | 0.22 | 21 + 32 % | 96 + 18 % | 97 + 40 % |
| **Tf** | **73.0623** | 73.0633 | **yes** | **yes** | **3.37** | **2.97** | **1922 + 52 %** | **570 + 12 %** | **646 + 13 %** |
| Tf | 76.0809 | - | yes | not present | - | - | - | - | - |
| Tf | 77.0362 | 77.0372 | yes | no | 0.37 | 0.31 | 56 + 7 % | 150 + 2 % | 181 + 27 % |
| Tf | 79.0515 | 79.0524 | yes | no | 0.34 | 0.27 | 100 + 14 % | 290 + 5 % | 369 + 39 % |
| Tf | 87.0785 | 87.0785 | yes | no | 1.51 | 1.09 | 343 + 18 % | 228 + 5 % | 314 + 14 % |
| Tf | 89.0577 | 89.0576 | yes | no | 1.31 | 1.19 | 1076 + 16 % | 823 + 5 % | 908 + 18 % |
| Tf | 92.0486 | 92.0491 | yes | no | 0.20 | 0.07 | 2 + 54 % | 12 + 7 % | 35 + 120 % |
| Tf | 96.0859 | 96.0869 | yes | no | 0.49 | 0.47 | 40 + 18 % | 82 + 3 % | 85 + 11 % |
| **Tf** | **100.0180** | 100.0167 | **yes** | **yes** | **5.12** | **2.79** | **61 + 28 %** | **12 + 29 %** | **22 + 76 %** |
| Tf | 103.0630 | - | yes | not present | - | - | - | - | - |
| Tf | 103.0729 | 103.0730 | yes | no | 0.64 | 0.60 | 329 + 3 % | 513 + 4 % | 552 + 21 % |
| Tf | 107.0463 | 107.0466 | yes | no | 0.29 | 0.29 | 233 + 31 % | 810 + 9 % | 794 + 30 % |
| Tf | 109.0516 | - | yes | not present | - | - | - | - | - |
| Tf | 109.0981 | 109.0999 | yes | no | 1.26 | 0.81 | 1755 + 48 % | 1390 + 15 % | 2172 + 40 % |
| Tf | 115.1081 | 115.1103 | yes | no | 0.98 | 0.66 | 231 + 35 % | 236 + 2 % | 348 + 30 % |
| Tf | 116.1120 | - | yes | not present | - | - | - | - | - |
| Tf | 123.1131 | 123.1145 | yes | no | 0.29 | 0.43 | 290 + 12 % | 988 + 11 % | 675 + 9 % |
| **Tf** | **127.1082** | 127.1100 | **yes** | **yes** | **12.17** | **8.27** | **14286 + 69 %** | **1174 + 6 %** | **1727 + 16 %** |
| **Tf** | **142.0290** | 142.0292 | **yes** | **yes** | **17.36** | **13.13** | **30 + 130 %** | **2 + 12 %** | **2 + 36 %** |
| Tf | 142.0474 | - | yes | not present | - | - | - | - | - |
| Tf | 183.1711 | 183.1735 | yes | no | 0.31 | 0.32 | 251 + 15 % | 818 + 16 % | 782 + 45 % |
| Tf | 189.1261 | 189.1250 | yes | no | 0.78 | 0.59 | 59 + 16 % | 76 + 3 % | 100 + 12 % |
